# Regulation of free heme and heme buffering in *Saccharomyces cerevisiae*

**DOI:** 10.1016/j.jbc.2026.113603

**Published:** 2026-09-23

**Authors:** Mathilda M. Willoughby, Yibo Fu, Courtney M. Moore, Yungeun Lee, Mauna S. Kareti, Liushu Wu, Li Zhang, Amit R. Reddi

**Affiliations:** 1 School of Chemistry and Biochemistry, Georgia Institute of Technology, Atlanta, Georgia, USA; 2 Department of Biological Sciences, The University of Texas at Dallas, Richardson, Texas, USA; 3 Parker Petit Institute for Bioengineering and Biosciences, Georgia Institute of Technology, Atlanta, Georgia, USA; 4 School of Biological Sciences, Georgia Institute of Technology, Atlanta, Georgia, USA

**Keywords:** heme homeostasis, heme trafficking, heme signaling, hydrogen peroxide, heme buffering, iron, glyceraldehyde phosphate dehydrogenase (GAPDH)

## Abstract

Heme is an essential but potentially cytotoxic metabolite. The requirement to tightly regulate heme led to the hypothesis that intracellular free heme is managed by a cellular buffering system. Despite its importance for understanding heme trafficking and signaling, the identity and buffering capacity of this buffer remain poorly understood. In this study, we utilized the model eukaryote *Saccharomyces cerevisiae* and genetically encoded fluorescent heme sensors to elucidate cellular heme buffering. Towards this end, we decreased hemoprotein expression by deleting the heme-regulated transcription factor Hap1, which controls the expression of several known heme-dependent proteins, or one of its highly abundant targets, the flavohemoglobin Yhb1, and measured the effect on free heme. We found that the change in free heme and heme sensor occupancy due to ablation of Hap1 and Yhb1 is consistent with the presence of a robust cellular heme buffering system. Results from equilibrium modeling and experimental measurements of free heme are consistent with the glycolytic enzyme glyceraldehyde phosphate dehydrogenase (GAPDH) serving a role as the primary cellular heme buffer. We estimate the buffer depth to be approximately ∼50 μM and a buffering capacity {Δ[Heme]_Total_/Δ[Heme]_Free_} of ∼1500. As part of our studies probing the effects of Hap1 on free heme, we found that hydrogen peroxide labilizes heme, establishing a direct link between reactive oxygen species (ROS) and cellular heme availability. Finally, we show that Hap1 is required for efficient mitochondrial heme export, albeit through an unknown mechanism. Together, our studies establish fundamental metrics and regulatory mechanisms for eukaryotic heme homeostasis.

Heme is an essential cofactor, signaling molecule, dietary source of iron, and flavor compound. As a cofactor, heme mediates several critical functions, including catalysis, electron transfer, and gas transport (1). As a signaling molecule, heme binding and/or dissociation can regulate enzyme activity or protein stability, thereby modulating various downstream biological processes (2, 3). Nutritionally, heme is a source of iron for many organisms, including humans, who are better able to absorb heme iron compared to non-heme iron (4). As a flavor compound, heme contributes to the “meaty” umami flavor of plant-based meat products (5). The biochemical, nutritional, and gastronomical roles of heme have led many labs to engineer microbes, including *Saccharomyces cerevisiae*, to mass produce heme or hemoproteins, for example, globins and cytochrome P_450_ enzymes, for various biotechnological applications, including as oxygen carriers for artificial blood, reagents for biocatalysis, and flavoring additives (6, 7, 8, 9, 10, 11, 12, 13, 14, 15, 16). However, due to the potential cytotoxicity of heme (17, 18, 19, 20), its use in metabolism and signaling, or as a commodity to be overproduced in cell factories, must be balanced by strategies that mitigate heme toxicity.

Heme trafficking and homeostatic mechanisms are under both thermodynamic and kinetic control. The consensus view is that cellular heme is buffered such that the concentration of unbound *free* heme is limiting (∼1–100 nM (21, 22, 23, 24)) and held relatively constant in the face of elevated heme synthesis or uptake (21, 23, 25, 26, 27). Such a model would necessitate that when there is a metabolic demand for heme, it can be transferred *via* exchange reactions down a thermodynamic gradient from sites that buffer heme to awaiting apo-hemoproteins. When the buffering capacity of a cell is exceeded, *free* heme can be enzymatically degraded *via* heme oxygenases (28, 29, 30), exported from cells (31, 32, 33), form insoluble aggregates (34, 35), or become sequestered in intracellular compartments or vesicles (36). Additionally, there are heme chaperones that make heme bioavailable by extracting it from heme-rich membranes and organelles, for example, transport and Golgi organization 2 (TANGO2) (37), or deliver heme to specific hemoprotein targets, for example*,* glyceraldehyde phosphate dehydrogenase (GAPDH) (38, 39) and heat shock protein 90 (Hsp90) (40).

In the context of this model, we can define several key species. Free heme is not bound to proteins or other biomacromolecules and coordinated by H_2_O/^−^OH or other anionic axial ligands, for example, Cl^−^. Exchange labile heme represents heme complexes that can reversibly bind and rapidly exchange heme on physiologically relevant time scales. Exchange inert heme represents heme bound to high-affinity and potentially buried heme-binding sites that have high barriers for exchange such that heme cannot readily dissociate or be transferred to other molecules. A heme buffer, in analogy to a proton buffer, would represent a heme complex that has exchange-labile binding sites that rapidly equilibrate between bound and free forms depending on total heme concentration, the expression of other heme-binding proteins, and their relative heme affinities. A heme chaperone binds and delivers heme to specific client proteins.

The identity, concentration, and affinities of molecules in what may be a polydisperse pool that buffers heme will dictate how effectively different cells and organisms limit free heme and its potential toxicity. Moreover, the nature of heme buffering ligands will have implications for the trafficking and mobilization of heme for use in metabolism and signaling. From a biotechnological perspective, the ability to engineer the properties of the buffer, including its concentration and/or composition, may limit heme toxicity when trying to overproduce heme and hemoproteins in cell factories. However, the heme buffering capacity of cells, as well as the nature of the molecules that constitute the heme buffer, are not well understood.

To elucidate cellular heme buffering capacity, we sought to generate cell lines with substantially diminished hemoprotein expression to probe its effects on free heme as assessed by genetically encoded heme sensors. In such a system, the extent to which free heme increases due to downregulation of hemoprotein expression would reflect the degree of heme buffering in cells. If heme buffering capacity is low, repression of hemoprotein expression should substantially increase free heme and heme sensor occupancy. Conversely, if heme buffering capacity is high, the downregulation of hemoproteins should result in only a modest increase in free heme and heme sensor occupancy because uncommitted *free* heme would be buffered. Thus, the magnitude by which heme sensor occupancy changes due to downregulation of hemoprotein expression will inform on the presence of a cellular heme buffer and its depth, *i.e.* concentration.

The genetically tractable, model unicellular eukaryote, *S. cerevisiae*, is an ideal host to manipulate heme protein expression without substantially perturbing total heme. Heme activator protein 1 (Hap1) is a transcription factor that regulates gene expression in both a heme-dependent and an independent manner (41, 42, 43). The function of Hap1 is to indirectly sense oxygen availability through heme, whose synthesis is oxygen-dependent, to regulate the expression of genes required for aerobic metabolism (3, 41, 44, 45, 46, 47, 48, 49, 50, 51). In the absence of heme, Hap1 forms a higher-order molecular complex with Hsp82, Ydj1, and other accessory factors (44, 46, 49). Heme binding to Hap1 disrupts this complex, allowing Hap1 dimerization, DNA binding, and subsequent transcriptional regulation (46). Hap1 contains cysteine-proline (CP) residues within several heme regulatory motifs (HRMs) that sense and bind heme to regulate its transcriptional activity (52, 53). Hap1 can act both as a promoter or repressor of gene expression (43). In the presence of oxygen, it can activate the expression of respiratory pathway genes, for example, cytochrome *c* isoforms (*CYC1*, *CYC7*) or a subunit of Complex III (*CYT1*), or antioxidant genes, for example, cytosolic catalase (*CTT1*), among many others, in a heme-dependent manner (43). In the absence of oxygen, Hap1 represses the expression of certain genes, including those for ergosterol biosynthesis, for example, hydroxymethylglutaryl-Coenzyme A reductase isoforms (*HMG1*, *HMG2*) and lanosterol 14-α-demethylase (*ERG11*), among others (43). Importantly, Hap1 positively regulates more than half of the most abundant known heme proteins in yeast (43) (Table 1). Thus, ablation of *HAP1* would allow for determining the impact of downregulating heme protein expression on sensor-detectable *free* heme and therefore heme buffering capacity.

**Table 1:** All Currently Known Established Heme-Dependent Proteins and Enzymes in Yeast

Systematic Name	StandardName	Descriptive Name	Heme Type (# of Hemes)	Median Molecules per Cell (57)	Localization	Hap1 Binding to Promoter (43, 112)	Fold Change in Expression (*hap1*Δ/WT) (43)
YGR234W	YHB1	Flavohemoglobin	*b* (1)	31,688	Cytosol	Y	0.64
YJR137C	MET5	Sulfite reductase subunit	siroheme	6101		N	0.93
YGR088W	CTT1	Catalase	*b* (1)	4910		Y	0.50
YMR073C	IRC21	Unknown function (contains Cytochrome *b*_5_ domain)	*b* (1)	2564		Y	0.35
YNL234W	YNL234W	Hemoglobin-like	*b* (1)	815		N	0.35
YJR078W	BNA2	Dioxygenase	*b* (1)	578		Y	0.36
YHR007C	ERG11	Cytochrome P_450_	*b* (1)	13,032	Endoplasmic Reticulum	Y	0.83
YMR015C	ERG5	Cytochrome P_450_	*b* (1)	8338		Y	0.59
YNL111C	CYB5	Cytochrome *b*_5_	*b* (1)	4468		Y	0.67
YMR272C	SCS7	Sphingolipid hydroxylase (contains Cytochrome *b*_5_ domain)	*b* (1)	3771		Y	0.74
YGL160W	YNO1	NADPH oxidase	*b* (2) (113)	1275		Y	1.03
YDR402C	DIT2	Cytochrome P_450_	*b* (1)	ND		N	0.96
YOR384W	FRE5	Ferric iron reductase	*b* (2) (114)	2212	Plasma Membrane	N	2.00
YLR214W	FRE1	Ferric iron reductase	*b* (2) (114)	1528		Y	0.50
YNR060W	FRE4	Ferric iron reductase	*b* (2) (114)	1141		Y	0.37
YOL152W	FRE7	Ferric iron reductase	*b* (2) (114)	120		N	0.04
YKL220C	FRE2	Ferric iron reductase	*b* (2) (114)	ND		N	1.16
YOR381W	FRE3	Ferric iron reductase	*b* (2) (114)	ND		N	0.92
YLL051C	FRE6	Ferric iron reductase	*b* (2) (114)	2026	Vacuole	Y	0.50
YKR066C	CCP1	Cytochrome *c* peroxidase	*b* (1)	7620	Mito IMS	N	0.40
YJR048W	CYC1	Cytochrome c	*c* (1)	7195		Y	0.07
YML054C	CYB2	Cytochrome *b*_2_	*b* (1)	4590		Y	1.49
YEL039C	CYC7	Cytochrome c	*c* (1)	3133		Y	0.082
YKL141W	SDH3	Heme binding subunit of Complex II	*b* (0.5)	5425	Mito IM	N	0.43
YOR065W	CYT1	Heme binding subunit of Complex III	*c* (1)	5162		Y	0.16
YDR178W	SDH4	Heme binding subunit of Complex II	*b* (0.5)	2850		N	0.83
Q0045	COX1	Heme binding subunit of Complex IV	*a* (2)	2440		N	0.46
Q0105	COB	Heme binding subunit of Complex III	*b* (2)	ND		N	0.53
YLR164W	SHH4	Alternative heme binding subunit of Complex II	*b* (0.5)	ND		N	1.50
YMR118C	SHH3	SDH3 homolog of unknown function	*b* (0.5)	ND		N	2.14
YDR256C	CTA1	Catalase	*b* (1)	1032	Mito Matrix	Y	0.85
YLR256W	HAP1	Heme regulated transcription factor	*b*	1450	Nucleus	Y	1.80

Herein, by integrating genetically encoded fluorescent heme sensors with molecular genetic and biochemical approaches, as well as equilibrium modeling, we found that ablation of Hap1 or the Hap1-target and most abundant hemoprotein in yeast, the flavohemoglobin Yhb1, resulted in elevations in heme sensor occupancy that are consistent with the ability of cells to strongly buffer intracellular heme. Our data are consistent with the glycolytic enzyme glyceraldehyde phosphate dehydrogenase (GAPDH) serving a role as a cellular heme buffer. We estimate that the depth of the heme buffer is ∼50 μM (equal to the concentration of GAPDH) and the heme-buffering capacity, that is, the change in total heme concentration required to change the free heme concentration {Δ[Heme]_Total_/Δ[Heme]_Free_}, is of the order of ∼1500. As part of our studies probing the effects of Hap1 on free heme, we also found that metabolically produced hydrogen peroxide leads to elevated free heme in cells, linking reactive oxygen species (ROS) and heme availability. Finally, Hap1 was found to be required for the efficient trafficking of heme out of the mitochondria, albeit through an unknown mechanism. Taken together, the studies herein establish key metrics and regulatory mechanisms for cellular heme buffering in a model for eukaryotic cell biology.

## Results

### Heme supply matches demand in *S. cerevisiae*

We sought to determine the extent to which heme is buffered in cells by depleting the expression of heme-dependent proteins. The relative increase in *free* heme due to attenuation of heme protein expression will inform the degree to which cells buffer heme. However, this requires that the total amount of heme is similar to the total amount of heme-binding proteins. If the amount of heme is much greater or less than the content of heme-dependent proteins, depletion of heme-binding proteins may not result in a substantial change in *free* uncommitted heme. Indeed, this is demonstrated by simulating the free heme concentration as a function of heme protein expression for three different heme concentrations (Fig. 1*A*). For a hypothetical hemoprotein with a dissociation constant for ferric heme (*K*_D_^III^) of 10 fM, changes in hemoprotein concentration result in relatively small changes in free heme when incubated with 10 or 0.1 equivalents of heme, but larger changes in free heme when incubated with 1 equivalent of heme (Fig. 1*A*).

**Figure 1:**
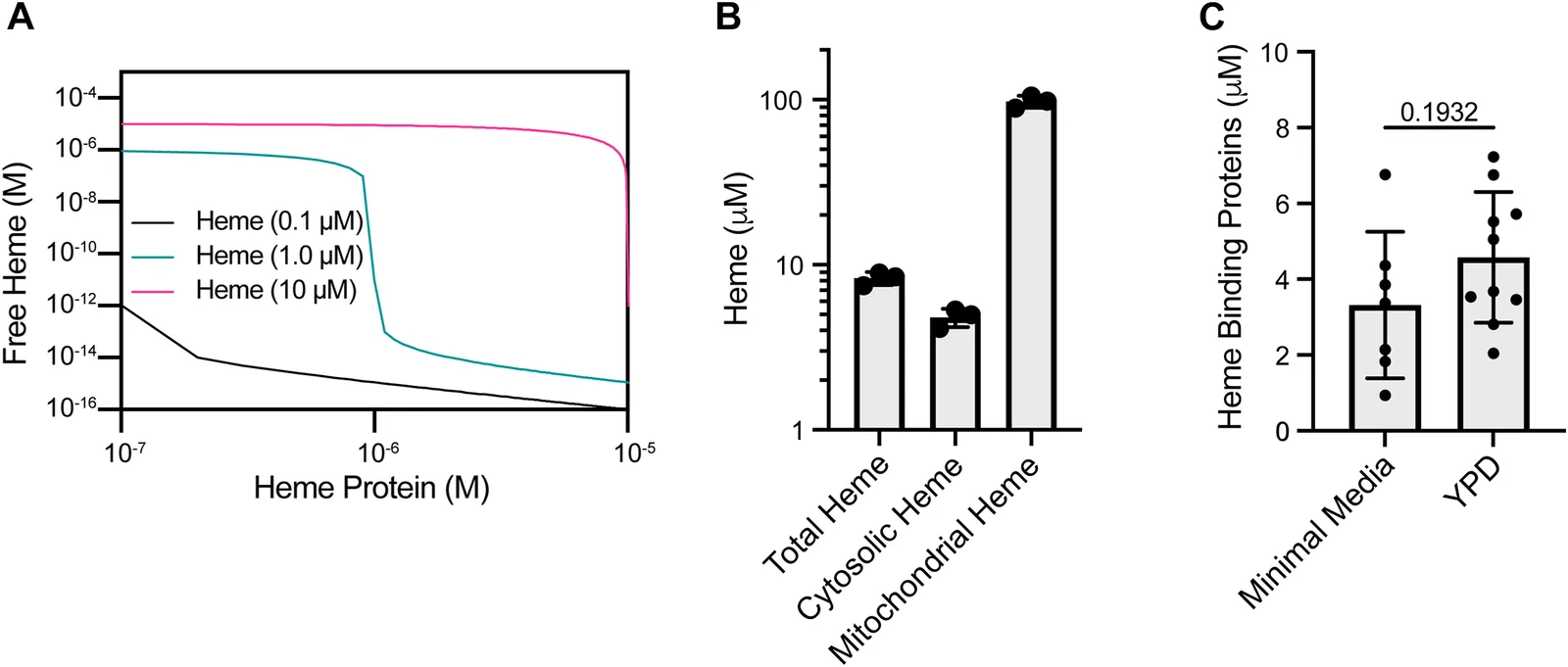
**Heme supply matches the demand for heme.***A*, simulation of free heme as a function of hemoprotein expression, assuming a *K*_D_ of 10 fM, for three different total heme concentrations; 0.1 μM (*black line*), 1.0 μM (*green line*), and 10 μM heme (*red line*). Simulations were generated using ChemEQL (89). *B*, measurements of heme in whole cells, cytosol, and mitochondria. *C*, the average and standard deviation in concentration of the known heme-dependent proteins listed in Table 1 as measured across 17 independent proteomics trials from reference (57). Proteomics trials are parsed according to whether cells were cultured in minimal media or YPD.

Consequently, we first sought to determine the content of cellular heme, that is, supply, relative to the copy numbers of all known heme-binding proteins, that is, demand. Table 1 lists all the known heme-dependent proteins in *S. cerevisiae*, along with the type of heme they bind, their copy numbers, and localization. Table 2 lists proteins involved in heme synthesis, degradation, or trafficking, which, due to the transient nature of their interactions with heme, are not considered to be substantially populated with heme. Since *c-*type and *a-*type hemes are derived from heme *b*, total heme can be approximated as the sum of these heme types. Siroheme-containing sulfite reductase was excluded from the count of heme-dependent proteins since siroheme is derived from the heme synthetic precursor uroporphyrinogen III and not heme *b*. Based on the sum of the median copy numbers of known heme-dependent proteins and their heme-polypeptide stoichiometries, the moles of heme-binding sites can be estimated to be 2.07 × 10^−19^, or a molarity of 4.1 μM if assuming a yeast cell volume of 50 fl (54) (Table S1). This value is remarkably similar to the total heme concentration in yeast measured from past studies, 1 to 10 μM (21, 55, 56), and the 8.3 μM value measured herein (Fig. 1*B*). Indeed, the range in the concentration of heme per cell roughly matches the standard deviation in concentration of heme-dependent proteins across 17 independent proteomics trials (Fig. 1*C*) (57). Together, these data suggest that cell-wide heme content approximately matches the metabolic demand for heme.

**Table 2:** Proteins required for maintaining heme homeostasis, including synthesis, degradation, trafficking, and buffering

Role in Heme Homeostasis	Systematic Name	Standard Name	Descriptive Name	Median Molecules per Cell	Localization	Hap1 Binding to Promoter (Y/N) (43, 112)	Fold-Change in Expression (*hap1*Δ/WT) (43)
Heme *b* Synthesis	YGR234W	HEM1	Aminolevulinic acid synthase (ALAS)	5028	Mito Matrix	N	1.04
	YHR007C	HEM2	Porphobilinogen synthase (PBGS)	8139	Cyto	N	0.70
	YJR048W	HEM3	hydroxymethylbilane synthase (HMBS)	5529	Cyto	Y	0.45
	YJR137C	HEM4	Uroporphyrinogen synthase (UROS)	2088	Cyto	N	0.35
	YML054C	HEM12	Uroporphyrinogen decarboxylase (UROD)	4391	Cyto	N	0.82
	YKR066C	HEM13	Coproporphyrinogen oxidase (CPOX)	5996	Mito IMS	N	1.74
	YMR015C	HEM14	Protoporphyrinogen oxidase (PPOX)	5048	Mito IM	N	0.98
	YPL170W	HEM15	Ferrochelatase (FECH)	8079	Mito IM	N	0.48
Heme *b* Degradation	YLR205C	HMX1	Heme oxygenase	1546	ER	Y	0.61
Heme Trafficking	YPL170W	DAP1	Damage resistance protein	3996	Mito OM + ER	Y	0.49
	YGR127W	TANGO2	Transport and Golgi Organization	1083	Mito	N	0.60
Heme Buffering and Trafficking	YJL052W	TDH1	Glyceraldehyde-3-phosphate dehydrogenase	80,420	Cyto	N	0.70
	YJR009C	TDH2		171,296		N	0.66
	YGR192C	TDH3		746,358		N	0.88
Heme *c* Synthesis	YAL039C	CYC3	Cytochrome *c* heme lyase	2357	Mito IM	Y	0.59
	YKL087C	CYT2	Cytochrome *c*_1_ heme lyase	1471	Mito IM	N	0.97
Heme *a* Synthesis	YPL172C	COX10	Heme *o* synthase	ND	Mito IM	N	0.54
	YER141W	COX15	Heme *a* synthase	6031	Mito IM	Y	0.11

Due to compartmentalization, we further assessed heme content in the cytosol and mitochondria relative to its use in these locales. ER, plasma membrane, and vacuolar membrane hemoproteins were counted as being “cytosolic” since the heme-binding domains of these proteins face the cytosolic side. Indeed, the heme chaperones, GAPDH and HSP90, which are localized in the cytosol, were previously found to mediate heme delivery to ER proteins like heme oxygenases (58) and cytochrome P_450_ enzymes (59), or the ER and plasma membrane integral membrane protein NADPH oxidase (NOX5) (60), suggesting that the heme binding sites of these proteins are accessible from the cytosolic side. Based on the sum of the median copy numbers of known heme-dependent proteins in the “cytosol”, the moles of heme-binding sites can be estimated to be 1.4 × 10^−19^, or a molarity of 4.1 μM if assuming a cytosolic volume for yeast of 35 fl, or ∼70% of the total volume of a cell, which is assumed to be 50 fl (54) (Tables S1 and S2). This value is similar to the 4.8 μM concentration of heme measured from cytosolic extracts of yeast (Fig. 1*B*). Thus, the number of moles of total heme in the cytosol roughly matches the demand for heme in this compartment.

Mitochondrial heme-dependent proteins reside in three distinct subcompartments—the mitochondrial inter-membrane space (IMS), the inner-membrane (IM), and the matrix. Based on the sum of the median copy numbers of known heme dependent proteins and their heme binding stoichiometries in the mitochondria, including the IMS, IM, and matrix, the moles of heme binding sites can be estimated to be 6.3 × 10^−20^, or a molarity of 125 μM if assuming a mitochondrial volume in yeast of 0.5 fl (54) (Table S1). This value is similar to the 97 μM concentration of heme (hemes *a* + *b* + *c*) measured from isolated mitochondria herein (Fig. 1*B*). Moreover, this range in values of total mitochondrial heme is similar to prior measurements in isolated mitochondria using electronic absorption spectroscopy that revealed total heme to be ∼114 μM; 14 μM heme *a*, 27 μM heme *b*, and 73 μM heme *c* (61). The concentrations of various heme types track with the concentrations and binding stoichiometries of proteins that require them; 16 μM heme *a* binding sites; 58 μM heme *b* binding sites; and 51 μM heme *c* binding sites (Tables 1 and S1). Taken together, the number of moles of total heme in the mitochondria is roughly matched to the demand for heme in this compartment as well.

Since the amount of heme is on the order of the copy number of all known heme-dependent proteins, it is expected that attenuating the expression of heme-dependent proteins will lead to changes in free heme and the magnitude of this change should reflect the heme buffering capacity of cells.

### Hap1 regulates heme protein expression

Prior chromatin immunoprecipitation (ChIP) experiments and microarray-based transcriptomics studies established the genome-wide targets of Hap1 regulation. Table 1 lists all the known heme-dependent proteins in *S. cerevisiae* and whether their expression is Hap1-dependent. 22 out of the 32 genes that encode known heme-dependent proteins have previously been found to be positively regulated by Hap1. Additionally, 15 of the 22 Hap1-regulated genes were previously found to have Hap1 bound or contain Hap1 consensus binding sequences. To confirm that the expression of Hap1 target genes is indeed influenced by ablation of Hap1, we probed the Hap1 or heme-dependent expression of a subset of these genes or their corresponding protein products.

In *S. cerevisiae* strain S288c, from which BY4741 is derived, the *HAP1* gene is partially inactivated due to insertion of the Ty1 transposon into the 3′ end of the open reading frame (ORF), resulting in the additional residues indicated in Figure 2*A* (62). Indeed, as shown in Figure 2, *B* and *C*, a pair of Hap1 transcriptional reporters revealed that *HAP1* deletion in BY4741 caused low Hap1 activity that was partially restored by introduction of the hypomorphic *HAP1*^TY1^ allele from S288c and fully restored by the functional *HAP1* allele from strains such as W303 or Sigma1278b. The transcriptional reporters employed included enhanced green fluorescent protein (eGFP) driven by the *CYC1* promoter (*CYC1*^pr^-eGFP), which is activated by Hap1 (Fig. 2*B*), or monomeric Katushka 2 (mKATE2) driven by the *HMG2* promoter (*HMG2*^pr^-mKATE2), which is repressed by Hap1 (Fig. 2*C*). As expected, based on prior work demonstrating that heme binds to and regulates Hap1, heme-depleted *hem1*Δ cells, which lack the first enzyme in the heme synthetic pathway, 5-aminolevulinic acid synthase (ALAS), phenocopied *hap1*Δ cells in terms of *CYC1* and *HMG2* expression.

**Figure 2:**
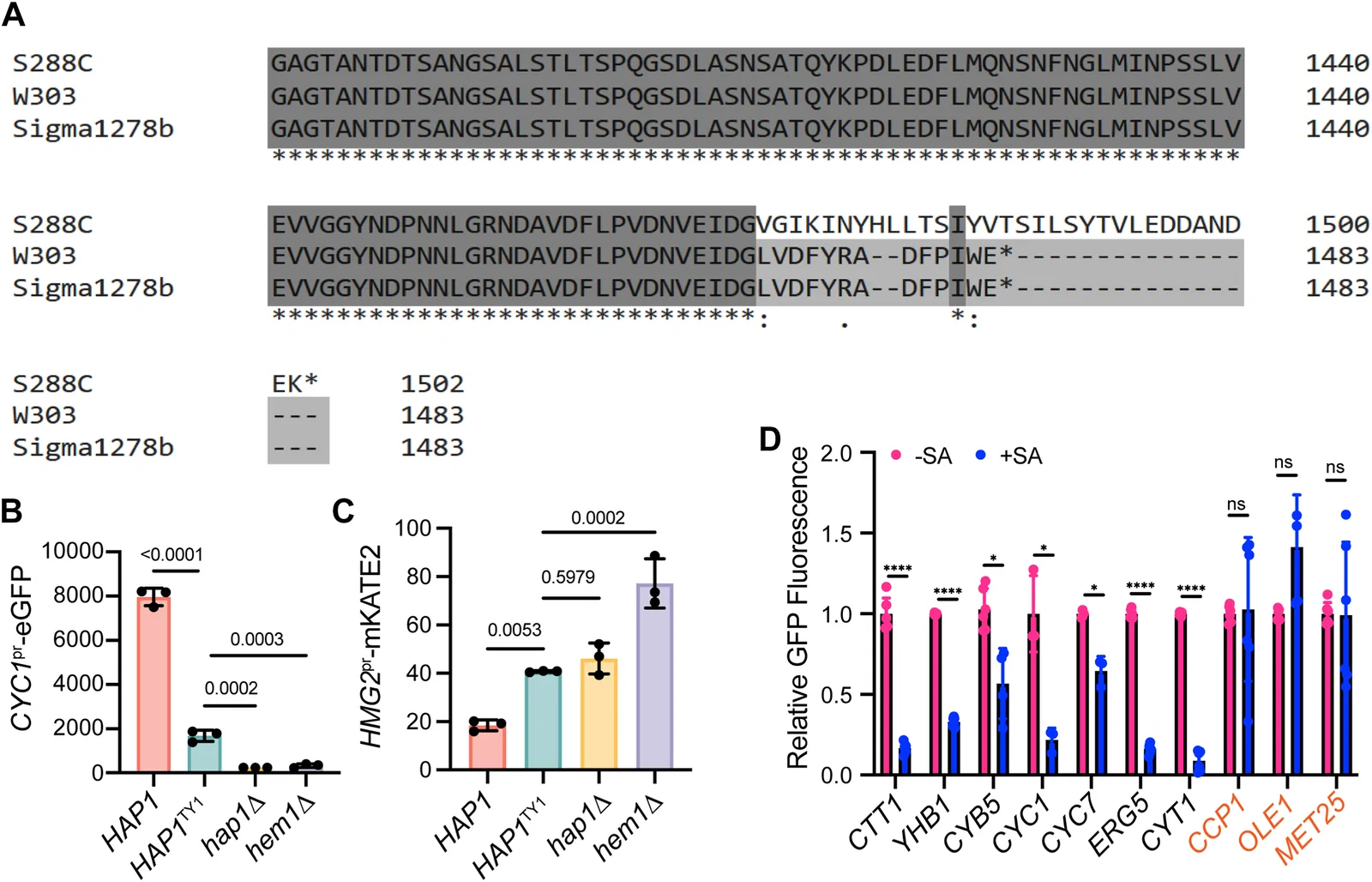
***Hap1*^TY1^ is hypomorphic and regulates the expression of several heme dependent proteins.***A*, sequence alignment of S288C Hap1^TY1^, which contains the TY1 transposon insertion in its C-terminal activation domain, and fully functional Hap1 from W303 and Sigma1278b *Saccharomyces cerevisiae* strains. *B-C*, Hap1 activity in the indicated strains as measured by a transcriptional reporter that uses eGFP driven by the *CYC1* promoter (*CYC1*^pr^-eGFP), which is a gene activated by Hap1, (*B*) and a reporter that uses mKATE2 driven by the *HMG2* promoter (*HMG2*^pr^-mKATE2), which is a gene that is repressed by Hap1 (*C*). *D*, heme-dependent expression of BY4741 strains expressing the indicated GFP-tagged proteins whose expression is known to be transcriptionally activated by Hap1 (*black*) or not (*orange*) as assessed by culturing cells without (*red*) or with (*blue*) 250 μM of the heme synthetic inhibitor succinylacetone (SA). The data in panels (*B*) and (*C*) represent the mean ± SD of independent triplicate cultures and the statistical significance (*p*-values) indicated was derived from an ordinary one-way ANOVA with Dunnett’s multiple comparisons test. The data in panel (*D*) represent the mean ± SD of 4 to 6 independent cultures and the statistical significance was derived from a paired *t* test between −/+ SA treatment for each strain. ∗*p* < 0.05, ∗∗∗∗*p* < 0.0001, n.s. *p* > 0.05.

To further validate that Hap1 regulates the hemoproteins listed in Table 1, the heme-dependent expression of selected C-terminal GFP-tagged hemoproteins driven by their native promoters was assessed. As shown in Figure 2*D*, the expression of known hemoprotein targets of Hap1 (Ctt1, Yhb1, Cyb5, Cyc1, Cyc7, Erg5, and Cyt1) were found to be heme-regulated, whereas the expression of proteins not known to be Hap1-regulated (Ccp1, Ole1, and Met25) were not heme-dependent (Table 1). Of note, the GFP-tagged strains assessed were in the background of BY4741, which contains the hypomorphic *HAP1*^TY1^ allele. Altogether, the results herein demonstrate that ablation of *HAP1* or heme, which activates Hap1, results in the downregulation of many of the most abundant hemoproteins, even in *HAP1*^TY1^ cells.

### Hap1 limits unbound heme

To determine if ablation of Hap1 activity leads to an increase in unbound heme due to the downregulation of the known heme dependent proteins, genetically encoded heme sensors HS1 and HS1-M7A were utilized. HS1 is a high-affinity heme sensor, having ferric and ferrous heme dissociation constant *K*_D_^III^ and K_D_^II^ values of 3 nM and 1 PM, respectively, and is quantitively saturated with heme in BY4741 containing the hypomorphic *HAP1*^*TY1*^ allele (Fig. 3*A*) (21). HS1-M7A is a moderate affinity sensor, having ferric and ferrous heme dissociation constant *K*_D_^III^ and K_D_^II^ values of 1 μM and 25 nM, respectively, and is 39% bound to heme in BY4741 containing the hypomorphic *HAP1*^*TY1*^ allele (Fig. 3*A*) (21). BY4741 *hap1*Δ cells exhibit very similar levels of heme sensor occupancy to *HAP1*^*TY1*^ expressing cells, presumably because *HAP1*^*TY1*^ is hypomorphic. On the other hand, expression of a fully functional *HAP1* allele without the TY1 insertion resulted in a substantial attenuation in HS1-M7A heme sensor occupancy, <1%. The Hap1-dependent effects on free heme observed in the cytosol were also recapitulated in the mitochondria (Fig. 3*B*) and nucleus (Fig. 3*C*) as assessed by mitochondrial and nuclear targeted heme sensors. The elevated free heme observed in cells lacking functional Hap1 is not due to elevated total heme content, as *hap1*Δ and *HAP1*^TY1^ cells had ∼27% less total heme than cells expressing the fully functional *HAP1* allele (Fig. 3*D*). The observation that functional Hap1 limits free heme was further validated by comparing heme sensor occupancy in two different yeast strains; one naturally containing the *HAP1*^TY1^ allele (BY4741) and one that naturally expresses the fully functional allele of *HAP1* (W303). Compared to W303, BY4741 exhibited greater levels of heme sensor heme occupancy and therefore free heme (Fig. 3*E*). The heme occupancy of HS1-M7A in BY4741 is 25% but in W303 it is < 5% (Fig. 3*E*). The heme occupancy of HS1 in BY4741 is 95% but in W303 it is 72%. Together, these results indicate that Hap1 limits unbound heme, presumably because of its transcriptional activation of several heme dependent proteins (Table 1).

**Figure 3:**
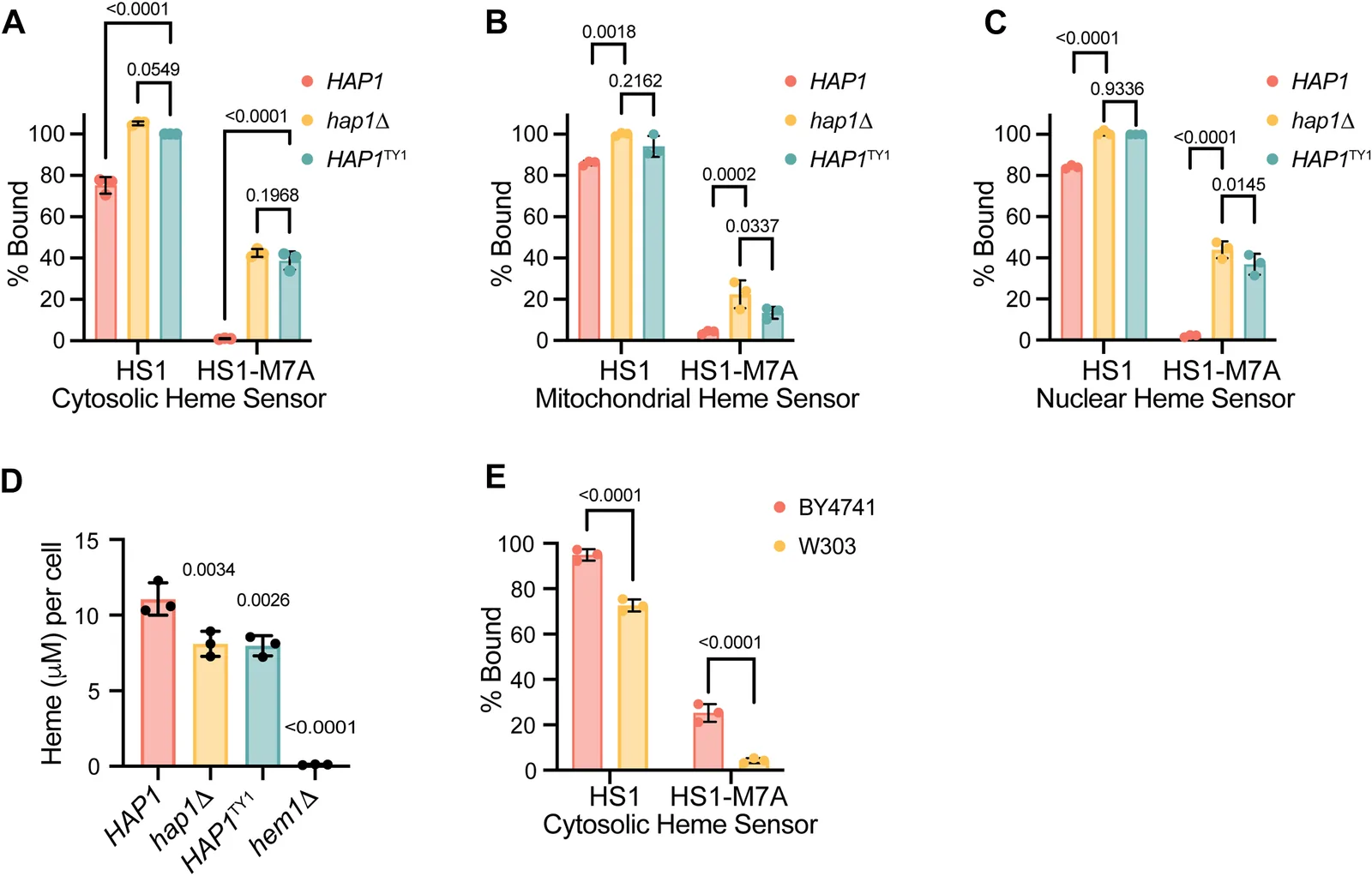
**Hap1 is required for limiting free heme in cells.***A–C*, heme occupancies of the heme sensors HS1 or HS1-M7A of the indicated strains with sensors targeted to the cytosol (*A*), mitochondria (*B*), or nucleus (*C*). *D*, Total heme levels in the indicated strains. *E*, Heme occupancies of the heme sensors HS1 or HS1-M7A in BY4741, which expresses *HAP1*^TY1^, and W303, which expresses *HAP1*. The data in panels (*A*–*C*) and (*E*) represent the mean ± SD of independent triplicate cultures and the statistical significance (*p*-values) indicated was derived from a two-way ANOVA with Šidák’s multiple comparisons test. The data in panel (*D*) represent the mean ± SD of independent triplicate cultures and the statistical significance (*p*-values) indicated relative to the *HAP1* strain was derived from an ordinary one-way ANOVA with Dunnett’s multiple comparisons test.

### Hap1 transcriptional activity is required to limit free heme

Hap1 contains seven HRMs (53), which have heme dissociation constants in the submicromolar to nanomolar regime (26, 63, 64, 65). Prior mutagenesis studies suggested that HRMs 1 to 6 potentially act to scavenge heme to maintain Hap1 activity when cellular heme concentration is low, whereas HRM7 is responsible for heme binding and mediating Hap1 heme responsiveness when the cellular heme concentration is higher (53). We therefore sought to determine if Hap1 regulates free heme indirectly due to its ability to transcriptionally control the expression of heme proteins or directly by binding to and sequestering heme. Towards this end, several *HAP1* mutant alleles with alterations in transcriptional activity or HRMs were examined for their influence on affecting heme sensor occupancy (Fig. 4*A*). The *hap1-18* allele, which has an S63R mutation in the DNA-binding zinc finger domain (Zn), and the *hap1*^*HK*^ allele, which lacks the activation (ACT) domain, both retain all the HRMs but lack transcriptional activity as previously assessed by probing *CYC1* expression (Fig. 4*A*) (42). Cells expressing Hap1^S63R^ (Fig. 4*B*) and Hap1^HK^ (Fig. 4*C*) exhibit HS1-M7A heme sensor occupancies that are elevated and similar to *hap1*Δ cells. Likewise, Hap1 truncation mutants, Δ444 to 1483 (HB) and Δ746 to 1483 (HG), which retain HRMs 1 to 6 but lack transcriptional activity, also exhibit *hap1*Δ-like elevated HS1-M7A heme sensor occupancies (Fig. 4*C*). On the other hand, hyperactive alleles of *HAP1*, including the Δheme *HAP1 allele (HAP1*^*Δheme*^), which lacks the repression module 1 (RPM1) and HRMs 1 to 6, and the H7-d1 *HAP1* allele *(HAP1*^*H7*−*d1*^), which retains HRMs 1 to 6 but lacks HRM7 and RPM2 (Fig. 4*A*), results in diminished HS1-M7A heme sensor occupancy and is similar to cells expressing functional WT *HAP1* (Fig. 4*C*). Altogether, these results indicate that Hap1 regulates free heme through its transcriptional activity, likely by controlling the expression of heme proteins, and not its capacity to directly bind heme *via* its HRMs.

**Figure 4:**
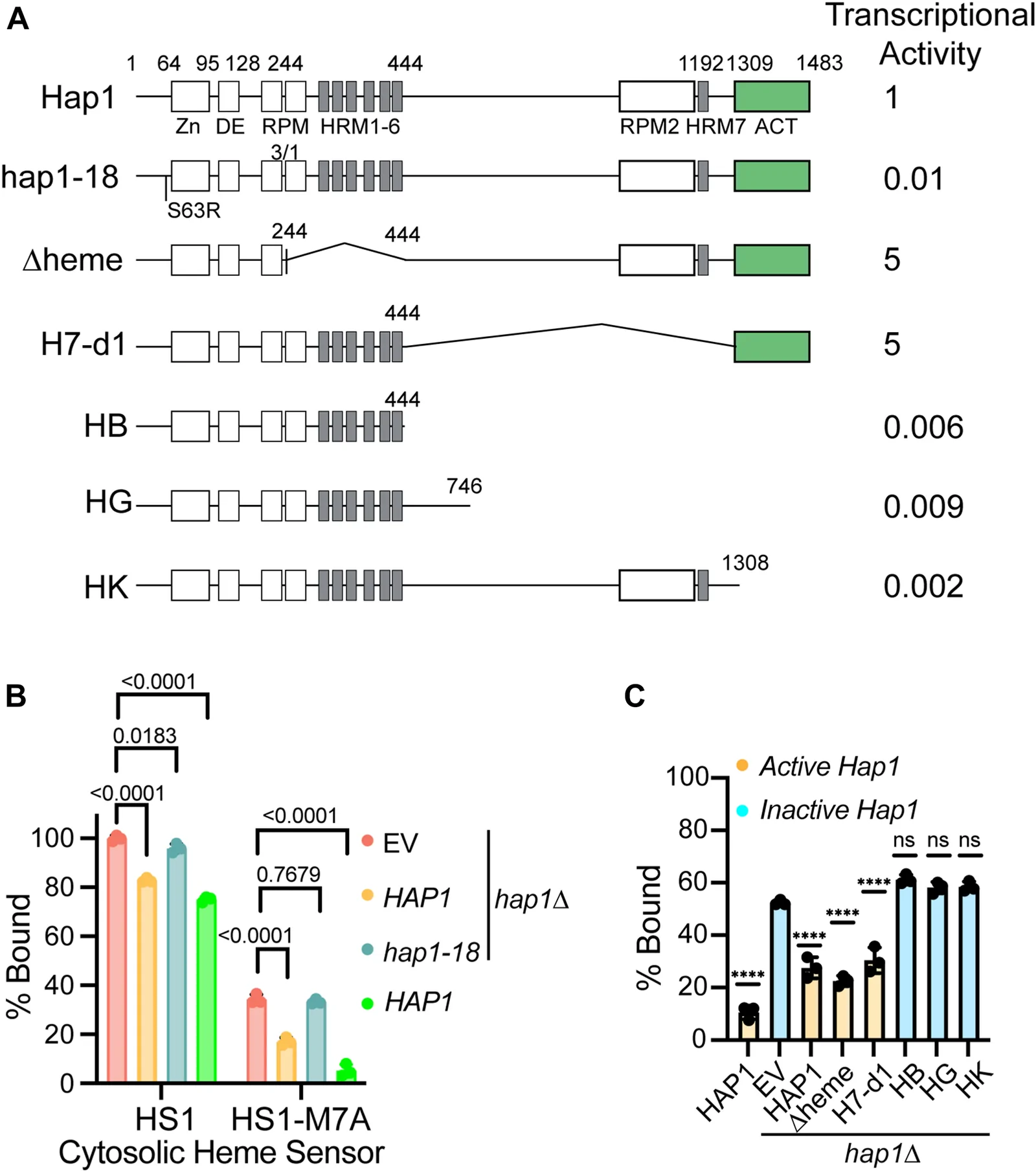
**Transcriptionally active Hap1, and not its heme regulatorymotifs, are required for limiting free heme in cells.***A*, schematic of the various *HAP1* alleles used in this study to determine if heme binding capacity and/or transcriptional activity is required to limit free heme in cells. The relative transcriptional activities of the indicated *HAP1* alleles were previously reported (42). Zn = Zinc Finger Domain; DE = Dimerization Element; RPM = Repression Module; HRM = Heme Regulatory Motif; ACT = Activation Domain. *B*, heme occupancies of the heme sensors HS1 or HS1-M7A in *hap1*Δ cells expressing empty vector (EV), *HAP1*, or *hap1-18* alleles on an episomal plasmid, or cells with an integrated copy of *HAP1. C*, heme occupancies of the heme sensor HS1-M7A in *hap1*Δ cells expressing empty vector (EV) or the transcriptionally inactive *hap1* alleles (HB, HG, and HK) (*blue*) or the transcriptionally active *HAP1* alleles (*HAP1*, Δheme, H7-d1) (*beige*), or in cells with an integrated copy of *HAP1* (*beige*). The data in panel (*B*) represent the mean ± SD of independent triplicate cultures and the statistical significance (*p*-values) indicated was derived from a two-way ANOVA with Dunnett’s multiple comparisons test. The data in panel (*C*) represent the mean ± SD of independent triplicate cultures and the statistical significance indicated relative to the *hap1*Δ EV strain was derived from an ordinary one-way ANOVA with Dunnett’s multiple comparisons test. ∗∗∗∗*p* < 0.0001, n.s. *p* > 0.05.

### Equilibrium modeling indicates cellular heme is buffered

The magnitude by which free heme increases due to ablation of heme-dependent proteins will indicate the extent to which heme is buffered in cells. Free heme, and therefore heme sensor occupancy, can be estimated using an equilibrium model that considers total heme, the concentration of known heme-dependent proteins (Table 1), and their heme dissociation constants, reduction potentials, and binding stoichiometries. To mitigate confounding factors due to compartmentalization, only “cytosolic” heme is considered, which also includes ER, plasma membrane, and vacuolar membrane proteins that can access cytosolic heme for the reasons discussed earlier (Table S2). The specific details and assumptions underlying the equilibrium model are outlined in the Supporting Information and Table S3, which lists the parameters used in the equilibrium model to predict free heme, including the “cytosolic” heme-dependent enzymes, their molar concentrations (assuming a cytosolic volume of 35 fl), and their assumed apparent heme dissociation constants and reduction potentials. Briefly, the model assumes that free heme is in the reduced state. If the resting oxidation state of heme in a protein is also reduced, free heme is governed by the ferrous heme dissociation constant. If the resting oxidation state of heme in a protein is oxidized, free heme is governed by the product of the equilibrium constants associated with heme reduction and ferrous heme dissociation (Supporting Information).

The free heme concentrations (Fig. 5*A*) and the HS1-M7A heme sensor occupancies (Fig. 5*B*) were calculated as a function of total heme based on the parameters in Table S3. To estimate free heme in *hap1*Δ cells, the copy numbers of heme proteins were adjusted based on the measured transcriptional changes assessed from past microarray-based transcriptomics studies (Tables 1 and S3) (43). In this case, the median hemoprotein concentration was multiplied by the fold change in gene expression due to deletion of *HAP1* (Table S3). In the absence of a cellular heme buffer, free heme is calculated to span ∼1 to 7 μM for total heme levels between 5 and 10 μM in both *HAP1*^+^ and *hap1* cells (Fig. 5*A*), which corresponds to HS1-M7A sensor heme occupancies > 97% (Fig. 5, *B* and *C*). However, experimentally, HS1-M7A heme sensor occupancies are generally < 35% in *HAP1*^+^ cells and 35% – 60% in *hap1* (*hap1*Δ or *HAP1*^TY1^) cells (Fig. 5*C*). Thus, when omitting a heme buffer in the equilibrium model, the predicted concentrations of free heme and sensor occupancy are much greater than what is observed in cells.

**Figure 5:**
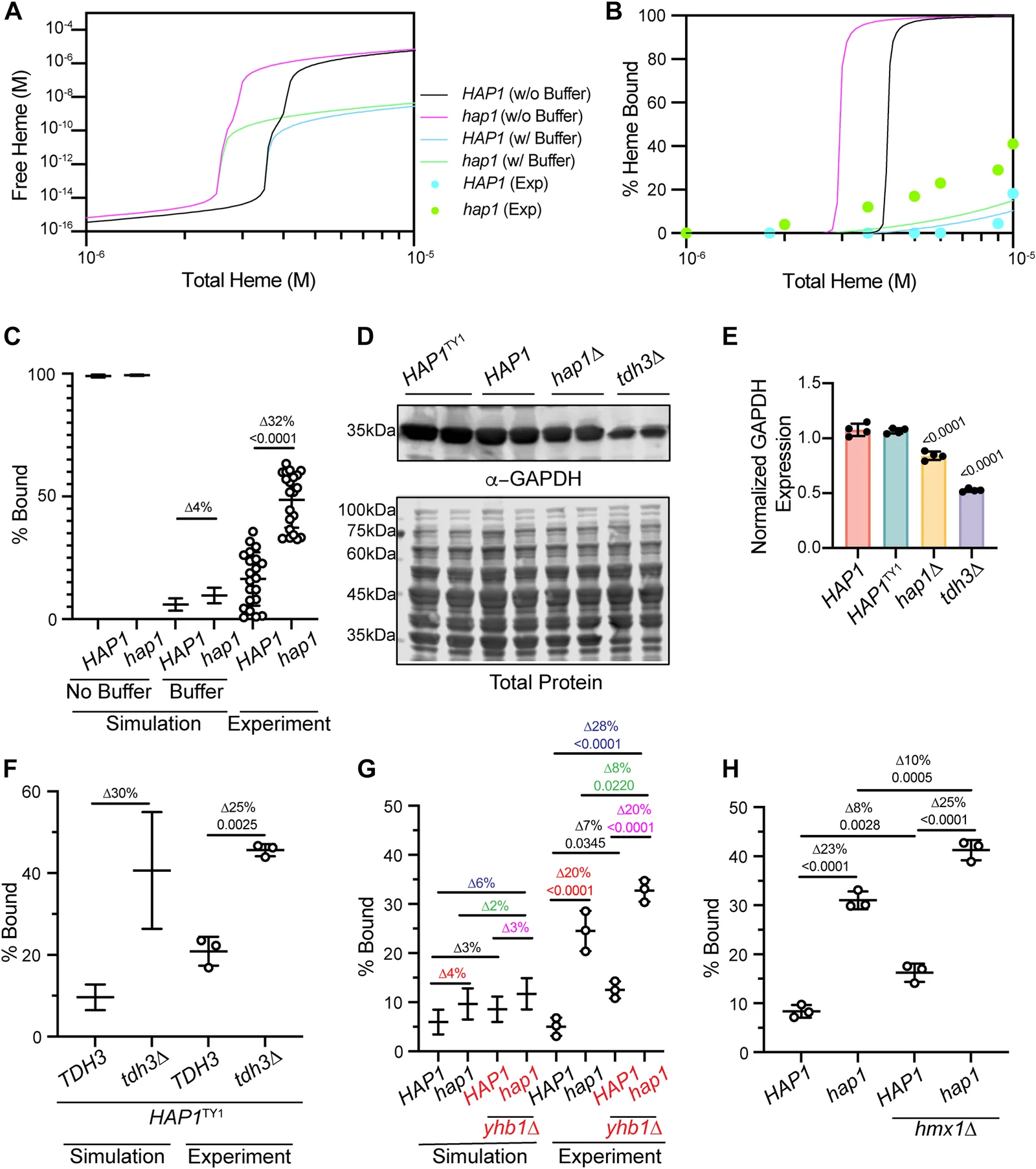
**Cytosolic heme is buffered and the cellular buffering system is consistent with GAPDH.***A-B*, simulation of free heme (*A*) and HS1-M7A heme sensor occupancy (*B*) as a function of total cytosolic heme based on the concentrations and assumed apparent heme dissociation constants of known “cytosolic” heme dependent proteins in *HAP1* and *hap1* cells with and without the GAPDH buffering system. In *panelB*, the *blue* and *green circles* represent measurements of HS1-M7A heme sensor occupancy in WT *HAP1* and *hap1*Δ cells, respectively, in cells titrated with the heme synthetic inhibitor succinylacetone (SA) to vary the intracellular heme content. Cells were cultured in SCE-LEU media overnight (∼15 h) with the following doses of SA: 500 μM, 250 μM, 100 μM, 50 μM, 25 μM, 10 μM, and 0 μM. *C*, a comparison between simulated and experimental HS1-M7A heme sensor occupancies in *HAP1* and *hap1* cells with and without the GAPDH heme buffer. The span in simulated heme sensor occupancies reflects the range in values associated with 5 to 10 μM total heme. *D–E*, a representative immunoblot (*D*) and quantification (*E*) probing changes in GAPDH expression in the indicated strains, along with a total protein stain as a loading control. Quantification of normalized GAPDH expression is derived from the ratio of GAPDH to total protein intensities. *F*, a comparison between simulated and experimental HS1-M7A heme sensor occupancies in WT and *tdh3*Δ mutants in the background of BY4741 *HAP1*^TY1^ cells. The span in simulated heme sensor occupancies reflects the range in values between 5 to 10 μM total heme. *G*, a comparison between simulated and experimental HS1-M7A heme sensor occupancies in WT and *yhb1*Δ mutants in the background of *HAP1* and *hap1* cells. The span in simulated heme sensor occupancies reflects the range in values between 5 to 10 μM total heme. *H*, HS1-M7A heme sensor occupancies in WT and *hmx1*Δ mutants in the background of *HAP1* and *hap1* cells. The data in *panel* (*B*) represent the mean of duplicate cultures and the variance in total heme and heme sensor occupancy measured for these samples is less than the diameter of the circles. The experimental data in *panel* (*C*) represents the mean ± SD of triplicate samples from seven independent trials (n = 21) and the statistical significance (*p*-value) indicated was derived from Welch’s two-tailed *t* test. The data in *panel E* represents the mean ± SD from duplicate samples in two independent trials (n = 4) and the statistical significance (*p*-value) indicated was derived from an ordinary one-way ANOVA with Dunnett’s multiple comparisons test. The data in panel (*F*) represents the mean ± SD of triplicate samples and the statistical significance (*p*-value) indicated was derived from Welch’s two-tailed *t* test. In data in panels (*G*) and (*H*) represent the mean ± SD of triplicate samples and the statistical significance (*p*-value) indicated was derived from an ordinary one-way ANOVA with Tukey’s multiple comparisons test. The underlying assumptions of the speciation model to calculate free heme and heme sensor occupancy in panels (*A–C*), and (*G*) are outlined in Table S3 and discussed in the Supporting Information.

### GAPDH buffers cellular heme

To assess the effects of a cellular heme buffer on free heme levels and sensor heme occupancy, glyceraldehyde phosphate dehydrogenase (GAPDH) was introduced into the equilibrium model to serve as the buffer. *S. cerevisiae* expresses three isoforms of GAPDH, including Tdh1, Tdh2, and Tdh3. It was previously found that ablation of *TDH3*, which encodes the most abundant GAPDH isoform, resulted in elevated free heme as measured by HS1-M7A (21). This led to the hypothesis that GAPDH may be an important constituent of the cellular heme buffer (21). We therefore parameterized the equilibrium model to include GAPDH. The cytosolic concentration of GAPDH is 47 μM in WT cells and 39 μM in *hap1*Δ cells) (Tables 2 and S3). GAPDH selectively binds ferric heme over ferrous heme (66), making heme reduction coupled to dissociation relevant for calculating free heme (Supporting Information).

In *HAP1*^+^ cells and in the presence of the GAPDH heme buffer, free heme is calculated to span 0.5 nM to 3 nM for total heme concentrations between 5 and 10 μM (Fig. 5*A*). This corresponds to HS1-M7A sensor heme occupancies between 2% and 10% (Fig. 5, *B* and *C*). In *hap1*Δ cells and in the presence of a cellular heme buffer, free heme is calculated to span 1 to 5 nM for heme levels between 5 and 10 μM (Fig. 5*A*), which corresponds to HS1-M7A heme sensor occupancies between 4% and 15% (Fig. 5, *B* and *C*). The heme sensor occupancy actually observed in *HAP1* and *hap1* cells is more consistent with the presence of a heme buffer (Fig. 5*C*). However, the observed magnitude increase in sensor heme occupancy due to ablation of *HAP1* (+32%) is much greater than that predicted (+4%) (Fig. 5*C*).

The greater-than-expected increase in free heme due to loss of Hap1 may be due to misparameterization of the equilibrium model. The concentration of the cellular heme buffer is a major determinant of free heme levels, and uncertainty in this parameter may give rise to the discrepancy between the predicted and observed difference in heme sensor occupancy between *HAP1* and *hap1*Δ cells. Assuming a total concentration of 5 μM cytosolic heme, the concentration of GAPDH would need to be reduced from ∼50 μM to < 10 μM in *hap1*Δ or *HAP1*^TY1^ cells to give rise to the 20% to 50% heme sensor occupancies observed (Fig. S1*A*). However, prior transcriptomic studies found that ablation of *HAP1* resulted in only a ∼20% decrease in GAPDH expression (43), which was considered in the equilibrium model used to calculate heme occupancy in *hap1*Δ cells (Fig. 5*C*). Immunoblotting for GAPDH likewise revealed a similar ∼20% reduction in expression due to deletion of *HAP1*. *HAP1-* and *HAP1TY1-expressing* cells have similar levels of GAPDH expression, and this is diminished by ∼20% in *hap1*Δ cells (Fig. 5, *D* and *E*), thereby confirming the assumed decrease in GAPDH expression due to deletion of *HAP1*.

To further examine the role of GAPDH as a cellular heme buffer, we deleted the gene encoding the most abundant GAPDH isoform in yeast, *TDH3*, and probed free heme levels. Deletion of *TDH3* results in a 50% decrease in total GAPDH protein expression (Fig. 5, *D* and *E*), which is similar to the expected ∼75% decrease in total GAPDH based on the copy numbers of Tdh1, Tdh2, and Tdh3 (Tables 2 and S3) and past studies indicating that Tdh3 contributed to ∼60% of total GAPDH activity in exponential-phase cells (67). *tdh3*Δ cells exhibited a 25% increase in sensor heme occupancy in the background of *HAP1*^TY1^-expressing BY4741 cells (Fig. 5*F*). The observed difference (Δ25%) in heme sensor heme occupancy between *HAP1*^TY1^ WT and *tdh3*Δ strains is similar to that predicted (Δ30%) based on an equilibrium model that considers a 75% decrease in GAPDH expression—∼40 μM GAPDH in WT cells and ∼10 μM GAPDH in *tdh3*Δ cells (Fig. 5*F*) (Table S3). Taken together, our results support a role for GAPDH in serving as the primary cellular heme buffer. However, it is unclear why Hap1 has a more pronounced effect on free heme than predicted. Given Hap1 is a transcription factor, there could be additional unknown Hap1-dependent factors that control free heme. Alternatively, the equilibrium model could be mis-parameterized or under-parameterized.

### Testing the equilibrium model predicting heme sensor occupancies

To further validate the equilibrium model, free heme as a function of total heme was experimentally assessed by measuring the changes in HS1-M7A heme occupancy and total heme due to heme depletion using the heme synthetic inhibitor succinylacetone (SA). Titration of SA over a broad range, between 0 and 500 μM, resulted in total heme levels that spanned ∼10 μM to ∼0.5 μM, respectively. In *HAP1*^+^ cells, experimental measurements of heme sensor occupancy as a function of total heme levels were very similar to that predicted based on the equilibrium model (Fig. 5*B*). However, in *hap1*Δ cells, the experimental measurements of heme sensor occupancy were considerably higher over a broad range of total heme concentrations (Fig. 5*B*). Together, these data suggest that the equilibrium model is appropriately parameterized to model *HAP1*^+^ cells, but that there are additional factors due to loss of *HAP1* not being accounted for in the model.

To determine if the equilibrium model was valid in a strain depleted of an abundant hemoprotein, but that still retained *HAP1*, heme sensor occupancy in the *yhb1*Δ mutant was assessed. Yeast flavohemoglobin, Yhb1, reversibly binds oxygen and contains heme- and flavin-binding domains (68, 69, 70). It is a nitric oxide oxidoreductase that plays a role in oxidative and nitrosative stress responses (69, 71). Yhb1 accounts for 40% of the known heme binding proteins (1.5 μM Yhb1 out of the 3.7 μM known “cytosolic” heme proteins) (Tables 1 and S3). Deletion of *YHB1* would be on par with ablation of *HAP1* in terms of attenuating hemoprotein expression. Based on the median hemoprotein concentrations and expected attenuation due to deletion of *YHB1* or *HAP1*, there would be an expected ∼40% or ∼32% reduction in hemoprotein expression, respectively (Table S3). The predicted 2 to 3% increase in heme sensor occupancy due to ablation of *YHB1* in *HAP1* and *hap1* cells is similar to the 7 to 8% increase actually observed (Fig. 5*G*), suggesting that the equilibrium model is generally appropriately parameterized. Thus, the greater than expected increase in free heme due to ablation of *HAP1* is likely due to additional regulatory mechanisms that extend beyond simple predictions based on the heme binding capacity of Hap1-regulated heme proteins.

The lack of data on heme binding thermodynamics of yeast hemoproteins (and hemoproteins more generally) is an obvious limitation in our modeling. Apparent heme dissociation constants for heme-dependent proteins, for example, cytochromes and globins, tend to be nanomolar or tighter, which was assumed in our model. On the other hand, heme-regulated proteins, for example, transcription factors and ion channels, tend to have weaker affinities, in the nM to μM regime. Weaker affinities ensure that heme-regulated proteins are fractionally occupied and/or heme binding is reversible in order to dynamically control protein activity. Even if the assumed apparent heme dissociation constants for heme-dependent proteins in our model were as weak as 100 nM, the prediction that heme sensor occupancies would be > 95% without a heme buffer and < 20% with GAPDH buffering heme would still hold true for total heme levels between 5 to 10 μM (Fig. S1*B*).

Another limitation in our model is the lack of understanding of the oxidation state of free unbound heme. Our model assumes that free heme is primarily reduced. If free heme were oxidized and ferric heme dissociation constants dictated free heme and HS1-M7A heme sensor occupancies, the predicted sensor occupancies would be 50% to 80% in *HAP1*^+^ (Fig. S1*C*), which is not observed. In *HAP1*^+^ cells, the observed HS1-M7A heme occupancies are often < ∼10% (Figs. 3, *A*, *E* and 4, *B*, *C*), but can be as high as 40% (Fig. 5*C*). Thus, the assumption that free heme in yeast is in its reduced state is supported by the observed heme sensor occupancies.

Overall, the assumptions related to the heme dissociation constants of heme-dependent proteins and the oxidation state of free heme are generally consistent with our observations of heme sensor occupancy in *HAP1*^+^ cells. However, the assumptions used to parameterize the equilibrium model fail to predict heme sensor occupancies in *hap1* cells.

### Heme oxygenase Hmx1, a target of Hap1, regulates free heme

*S. cerevisiae* encodes a heme oxygenase enzyme, *Hmx1*, that degrades heme into biliverdin (72). *HMX1* is a transcriptional target of Hap1 and is downregulated in *hap1*Δ cells (Table 2). Therefore, it is possible that the greater-than-anticipated increase in free heme due to ablation of *HAP1* is due to decreased Hmx1 expression and activity. However, while *hmx1*Δ cells exhibit an increase in free heme and heme sensor occupancy consistent with the inability to degrade heme, the magnitude of the increase does not phenocopy *hap1*Δ cells (Fig. 5*H*). Deletion of *HMX1* results in an 8 to 10% increase in heme sensor occupancy (Fig. 5*H*), whereas ablation of *HAP1* results in as much as a ∼30% increase (Fig. 5, *C*, *G* and *H*). This suggests that there are additional Hap1-dependent regulatory mechanisms that control free heme beyond heme degradation.

### Hydrogen peroxide regulates heme availability

Catalase is a hydrogen peroxide (H_2_O_2_) detoxifying enzyme. *S. cerevisiae* expresses both a cytosolic catalase, Ctt1, and a mitochondrial and peroxisomal catalase, Cta1. In exponential phase cells and in the culture conditions employed herein, Ctt1 accounts for the bulk of catalase activity (>95%) (73). Ctt1 is a target of Hap1 (Table 1), and its expression (Fig. 6, *A* and *B*) and activity (Fig. 6, *A* and *B*) are downregulated when *HAP1* is ablated. Ctt1 accounts for ∼6% of known heme-binding proteins (0.2 μM Ctt1 out of the 3.7 μM known “cytosolic” heme proteins) (Tables 1 and S1). If only considering the cytosolic concentration and heme-binding potential of catalase, deletion of *CTT1* is only expected to result in a < 0.5% increase in heme sensor occupancy for total heme concentrations between 5 and 10 μM (Fig. 6*D*). However, the observed magnitude increase in sensor heme occupancy due to deletion of *CTT1* is much greater (+29%), and very similar to the effects of deleting *HAP1* (+26%) (Fig. 6*D*). These data suggest that the greater-than-expected increase in free heme due to ablation of *HAP1* is due to attenuation of Ctt1 expression. Indeed, deletion of *CTT1* in the background of *hap1*Δ cells results in a more subdued increase in heme occupancy (+18%) compared to its deletion in *HAP1* cells (+29%) (Fig. 6*D*), consistent with attenuated Ctt1 expression and activity in the former (Fig. 6, *A*–*C*).

**Figure 6:**
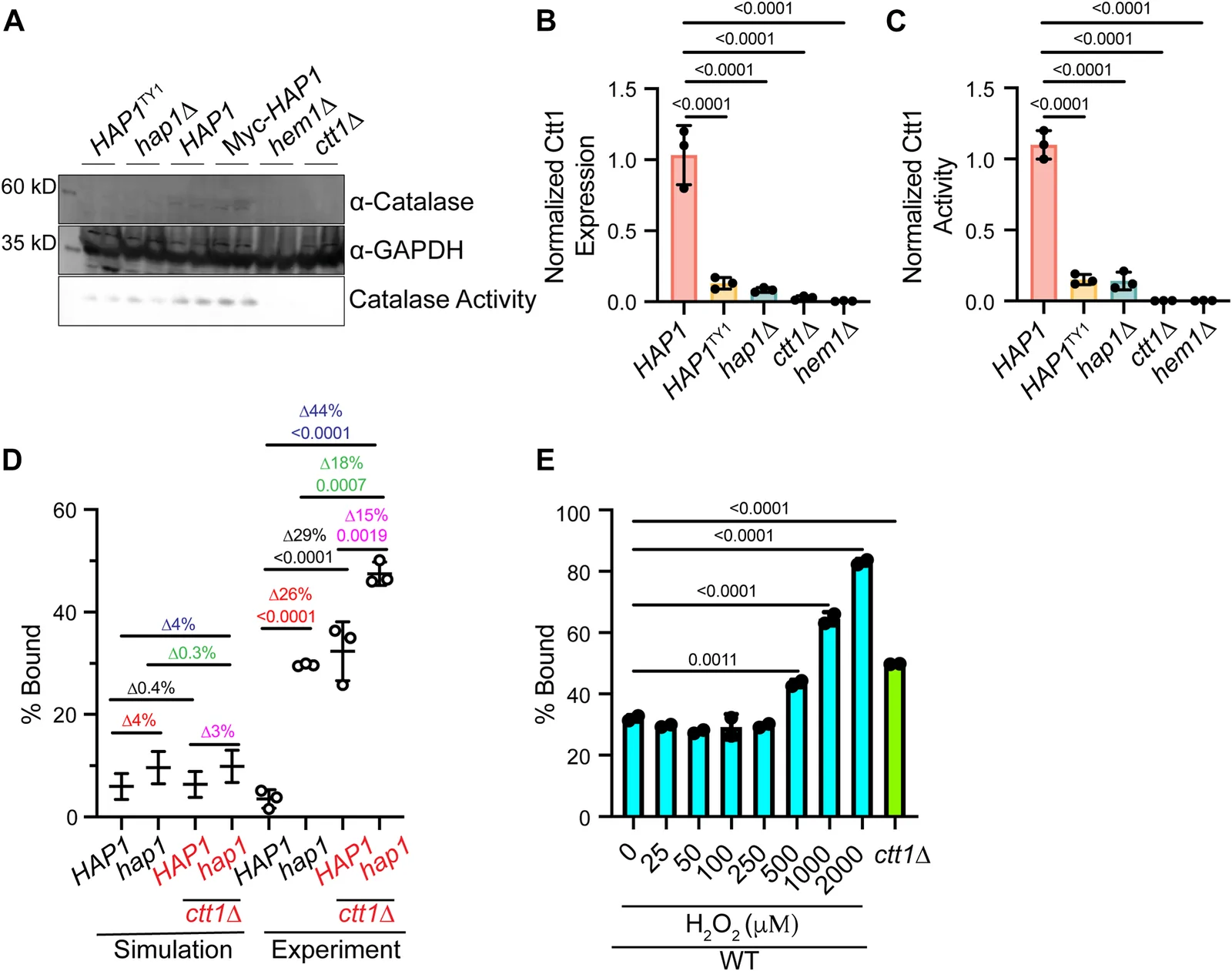
**Hydrogen peroxide labilizes and regulates free heme.***A–C*, representative immunoblot probing changes in catalase (Ctt1) expression and activity, along with a GAPDH loading control (*A*), and quantification of normalized Ctt1 expression (*B*) and activity (*C*) in the indicated strains. Quantification of normalized Ctt1 expression is derived from the ratio of the Ctt1 and GAPDH band intensities. While GAPDH expression does decrease by ∼20% in *hap1* strains (Fig. 5, *D* and *E*), the change in Ctt1 expression is much larger than changes in GAPDH. Quantification of normalized Ctt1 activity is derived from the ratio of the band intensity of each of the samples to the average intensity observed for *HAP1* cells from the native gel zymogram. *D*, a comparison between simulated and experimental HS1-M7A heme sensor occupancies in WT and *ctt1*Δ cells with or without *HAP1*. The span in simulated heme sensor occupancies reflects the range in values associated with 5 to 10 μM total heme. *E*, HS1-M7A heme sensor occupancies in BY4741 WT cells cultured with the indicated concentration of H_2_O_2_ for 1.5 h, and in *ctt1*Δ cells. The data in panels (*B*), (*C*), and (*E*) represent the mean ± SD of triplicate cultures and the statistical significance (*p*-value) indicated was derived from an ordinary one-way ANOVA with Dunnett’s multiple comparisons test. The data in *panel D* represents the mean ± SD from triplicate cultures and the statistical significance (*p*-value) indicated was derived from an ordinary one-way ANOVA with Tukey’s multiple comparisons test. The underlying assumptions of the speciation model to calculate free heme and heme sensor occupancy in *panel D* are outlined in Table S3 and discussed in Supporting Information.

Deletion of *CTT1* results in elevated concentrations of H_2_O_2_ (73). To determine if H_2_O_2_ is the key molecular species responsible for the increase in elevated free heme, HS1-M7A heme sensor occupancy was measured in cells that were cultured with varying concentrations of H_2_O_2_ (0–2 mM) for 1.5 h. Indeed, H_2_O_2_ concentrations between 0.5 and 2 mM resulted in substantial and statistically significant increases in heme occupancy, from ∼30% (without H_2_O_2_) to as much as ∼80% (with 2 mM H_2_O_2_) (Fig. 6*E*). Taken together, the major contributor toward the elevated free heme in *hap1*Δ cells is endogenous H_2_O_2_, which is increased due to diminished Ctt1 expression.

### Hap1 regulates heme trafficking kinetics

We next investigated whether Hap1 affects inter-compartmental heme trafficking rates. We previously developed a heme trafficking dynamics assay in which the rate of heme trafficking from the mitochondrial inner-membrane (location of the terminal heme synthetic enzyme, ferrochelatase) to the cytosol, nucleus, and mitochondrial matrix is monitored by probing the change in heme occupancy of sensors targeted to these locales. Toward this end, heme synthesis is first blocked using succinylacetone (SA), an inhibitor of the second enzyme in the heme synthetic pathway, porphobilinogen synthase (PBGS), and then re-initiated by removing the inhibitor from the media. Heme binding to nuclear, mitochondrial matrix, or cytosolic-targeted HS1 is then monitored over time as heme synthesis is reinitiated upon removal of SA. Using this assay, it was found that heme trafficking from the mitochondrial inner-membrane to the cytosol (Fig. 7*A*) and nucleus (Fig. 7*B*) is substantially attenuated in *hap1*Δ cells. On the other hand, trafficking into the mitochondrial matrix is not affected (Fig. 7*C*). The defects in heme trafficking rates associated with loss of *HAP1* are not due to changes in heme synthesis as both *HAP1*^+^ and *hap1*Δ cells produce heme at similar rates (Fig. 7*D*).

**Figure 7:**
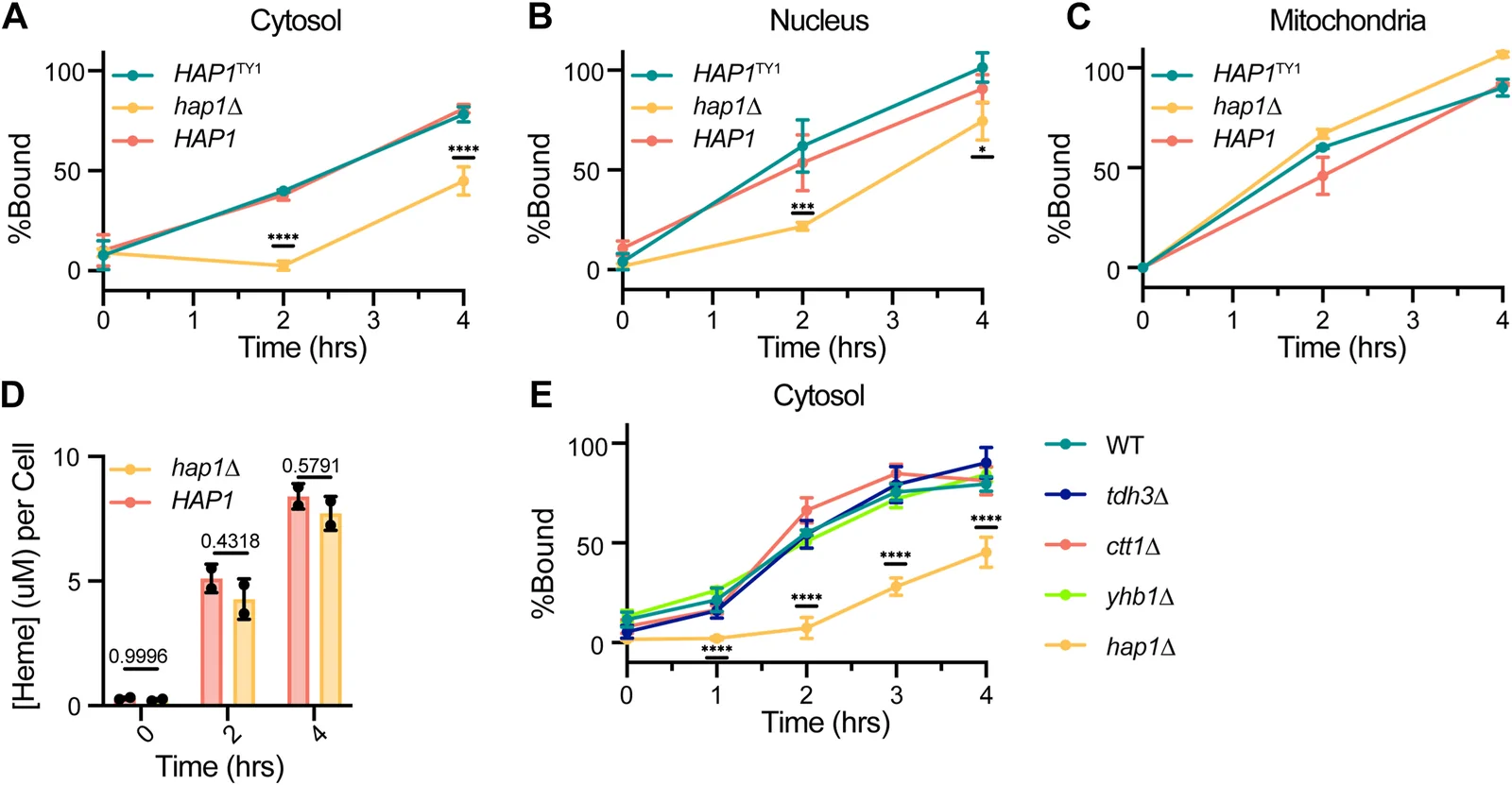
**Hap1 regulates heme trafficking dynamics.***A-C*, the indicated strains expressing HS1 in the cytosol (*A*), nucleus (*B*), or mitochondrial matrix (*C*) were depleted of heme using 500 μM succinylacetone (SA), a heme biosynthetic inhibitor, and, upon the re-initiation of heme synthesis, the rates of heme trafficking to the indicated subcellular locations were monitored by measuring the time-dependent change in HS1 heme sensor occupancy. *D*, the indicated strains were depleted of heme using 500 μM SA and, upon the re-initiation of heme synthesis, heme content was measured at the indicated time points to reveal an apparent rate of heme synthesis. (*E*) The rate of heme trafficking between the mitochondria and cytosol was measured in the indicated strains as described for *panel A*. In data in panels *A – C* and *E* represent the mean ± SD of triplicate cultures and the statistical significance indicated relative to the *HAP1* strain (*A-C*) or WT (*E*) was derived from a two-way ANOVA with Dunnett’s multiple comparisons test. ∗∗∗∗*p* < 0.0001, ∗∗∗*p* < 0.001, ∗*p* < 0.05. The data in *panel D* represents the mean ± SD of duplicate cultures and the statistical significance (*p*-value) indicated was derived from a two-way ANOVA with Šidák’s multiple comparisons test.

Since GAPDH (Fig. 5*F*), catalase (Fig. 6*D*), and Yhb1 (Fig. 5*G*) all affect free heme and are positively regulated by Hap1 (Tables 1 and 2), we sought to individually probe their roles in regulating heme trafficking rates. However, *tdh3*Δ, *ctt1*Δ, and *yhb1*Δ all exhibit WT-like trafficking rates (Fig. 7*E*). Taken together, Hap1 regulates heme trafficking rates but in a manner not dependent on changes in the concentration of the cellular heme buffer (GAPDH), the most abundant yeast hemoprotein (Yhb1), or hydrogen peroxide (*ctt1*Δ).

## Discussion

Heme is an essential but potentially cytotoxic metabolite. Consequently, cells and organisms are challenged to tightly regulate heme concentration and bioavailability. Arguably, the best understood mechanisms to control heme availability are the regulation of heme synthesis and degradation. More recently, additional paradigms for regulating cellular heme availability have emerged, including chaperone-mediated heme trafficking to specific client proteins, cellular heme export and import, heme sequestration and/or storage compartments, and heme buffering. Amongst all the currently known heme homeostatic mechanisms, perhaps the least understood is cellular heme buffering. Our current work sought to establish key attributes of the heme buffer, including its identity, concentration, and the cellular heme buffering capacity. Towards this end, free heme, as detected by the genetically encoded heme sensor, HS1-M7A, was assessed in WT and hemoprotein-deficient *hap1*Δ yeast strains and compared to predicted values of free heme in the absence or presence of the putative GAPDH heme buffering system. Altogether, our results strongly suggest that free heme is indeed buffered; GAPDH is a cellular buffer; endogenous hydrogen peroxide is a key determinant of heme labilization and accessibility; and Hap1 controls heme trafficking dynamics, albeit through still-unknown mechanisms.

In measuring heme, it was assumed that the heme sensor equilibrates with the buffered free heme being measured and that the sensor does not substantially perturb heme homeostasis by altering heme synthesis or degradation or acting as a heme sink. To the degree that these assumptions are not met, there would be inaccuracies in the estimates of free heme and buffering capacity. Indeed, we previously found that titration of HS1 in the ∼0.1 to ∼1 μM regime, which is within the range employed in experiments, does not affect heme sensor occupancy, cell growth, intracellular heme content or various heme-dependent processes (21). Moreover, previous equilibrium modeling studies found that heme sensor expression would not significantly perturb the observed levels of free heme at the concentrations employed (23, 26). Thus, there is no obvious indication that sensor expression itself substantially impacts estimates of sensor occupancy, free heme, or the conclusions drawn herein.

Our data on heme buffering in yeast is most consistent with free heme being reduced (Fig. S1*C*), which may not always be the case in every cell line and/or organism. For instance, using heme oxygenase-2 (HO-2) as a ferric heme-specific heme chelator, it was found that free heme in human embryonic kidney HEK293 cells is likely oxidized (28). Cellular heme speciation and redox environment will ultimately dictate the oxidation state of free heme and heme bound to labile sites, as well as the redox equilibration between the two.

Deletion of *TDH3* was previously shown to elevate free heme and, at the time, it was proposed that GAPDH could serve a function in heme buffering (21). In this study, by leveraging several independent proteomics studies that established the copy numbers of known heme-binding proteins in yeast (57), equilibrium modeling was conducted to estimate free heme and sensor heme occupancy to more quantitatively validate that GAPDH could serve as the primary heme buffering system in cells. Indeed, parameterization of an equilibrium model with a heme buffer consistent with the properties of GAPDH in terms of concentration and heme binding affinity predicted free heme levels to those experimentally observed in WT cells (Fig. 5*B*).

It was previously shown that GAPDH binds heme using a conserved histidine residue at the interprotomer interface of the GAPDH tetramer (39). Each GAPDH monomer binds heme in a 1:1 stoichiometry, with ferric and ferrous heme dissociation constants of 144 nM and 44 μM, respectively (66). The selectivity of GAPDH for ferric heme may support a redox-linked mechanism for heme buffering in which oxidation or reduction is coupled to heme binding to or dissociation from GAPDH, respectively (66). With heme bound at the protomer interface, it is also possible that the GAPDH oligomerization state is coupled to heme binding and/or dissociation. The dynamic regulation of oxidizing or reducing equivalents that alter heme redox state or GAPDH oligomerization may further tune the heme buffering capacity of cells in response to various physiological cues.

It is unclear if the heme buffering function of GAPDH is distinct from or explains its apparent heme chaperoning activity (38, 39). As a buffer, the abundance and heme affinity of GAPDH limit the concentration of free heme, especially in the face of excess heme. Reversible heme binding to GAPDH would ensure that, as heme is utilized by other heme-dependent enzymes and pathways, heme would dissociate from GAPDH and be acquired by client proteins or be transferred *via* exchange reactions down a thermodynamic gradient to client hemoproteins or other intermediaries. Metallochaperones, on the other hand, are specialized carriers that drive metallocofactor insertion into one or a small subset of client proteins in a manner that requires specific protein-protein interactions. Unlike buffers, metallochaperones need not be present at high concentrations and can bind cofactors irreversibly, with client protein interactions and/or coupling to energy-generating processes, *e.g.* nucleotide triphosphate hydrolysis, driving structural changes that initiate cofactor transfer. GAPDH was previously found to regulate heme insertion into several client proteins, including nitric oxide synthase (74), NADPH oxidase (60), IDO/TDO (75), Cytochrome P_450_ enzymes (59), and hemoglobin (40, 76). The relatively large substrate scope may suggest that GAPDH is not a chaperone *per se*, but rather a buffer that ensures heme is solubilized, preventing its aggregation and precipitation, and made available to client proteins. Identification of hemoproteins that are not GAPDH-dependent or elucidation of structural features that account for the large substrate scope of GAPDH may better resolve the function of GAPDH as a heme buffer *versus* a chaperone, albeit these functions are not mutually exclusive.

Assuming GAPDH is the primary heme buffer, it can be estimated that the depth or concentration of the buffer is equal to the concentration of GAPDH, ∼ 50 μM. At total heme levels above the concentration of known heme-requiring proteins in the “cytosol,” > 4.1 μM, but below the depth of the buffer (<∼50 μM), the heme buffering capacity, *i.e.* the change in total heme required to change free heme (Δ[Heme]_Total_/(Δ[Heme]_Free_), is on the order of ∼1500. (Fig. 5*A*). By contrast, weak buffering would approach a buffering capacity of 1. When overproducing heme and/or hemoproteins in yeast or other microbes for biotechnological purposes, one can now consider rationally tuning the heme buffering capacity of cells by altering GAPDH expression to mitigate heme toxicity.

The peroxide-induced increase in free heme that we observe is reminiscent of our past work demonstrating that nitric oxide (NO) can labilize heme, placing both NO and peroxide as key mediators of intracellular heme mobilization (21). Moreover, the observation that peroxide can labilize heme is consistent with prior work that demonstrated H_2_O_2_ can trigger heme transfer between proteins (77). In this case, H_2_O_2_ labilizes heme in cytochrome *c* peroxidase (Ccp1) by oxidizing a heme-iron-coordinating His ligand, leading to heme dissociation and transfer to mitochondrial catalase Cta1 (77). Inspired by this work, we propose that peroxide may labilize heme from other proteins by oxidizing heme-coordinating Cys or His residues, leading to heme dissociation and elevated levels of free heme. Alternatively, peroxide might alter the heme buffering capacity of cells by modulating GAPDH expression or inducing oxidation of the heme-coordinating His of GAPDH. The mechanisms underlying the peroxide-mediated labilization of cell-wide heme remains to be elucidated.

In retrospect, simple models to predict free heme and heme buffering capacity based on just the heme binding capacity of proteins are naïve. Endogenous reactive metabolites such as H_2_O_2_ or NO will have a profound effect on heme labilization and sensor heme occupancy. On the other hand, deviations from simple equilibrium binding models can inform on more complex forms of heme regulation, which was the basis for the discovery herein that endogenous peroxide is an important determinant of free heme.

One limitation of our studies is that we relied on the use of deletion mutants to perturb the expression of heme binding proteins. There may be unforeseen adaptive responses to such mutations that are not explicitly being considered. For instance, the unexpected increase in free heme due to ablation of Hap1 or Ctt1, which we attributed to elevated peroxide, may also be due to changes in protein expression and metabolism that are not being accounted for. The use of regulatable promoters or induced degradation of candidate proteins using conditional degrons may limit longer term adaptive responses.

Our data now reveal a complex feedback regulatory circuit in which heme regulates the response to peroxide stress. Elevated peroxide stress may lead to increased buffered free heme that can further activate Hap1 to upregulate catalase expression to scavenge peroxide. Our data also now suggest that, in addition to NO, peroxide can serve as a dynamic signal to control heme mobilization to regulate targets of heme signaling.

The mechanisms underlying the dynamic trafficking of heme from the matrix side of the mitochondrial inner-membrane (IM), where it is synthesized, to other subcellular locales is not well understood. Herein, we found that Hap1 is required to maintain optimum rates of heme trafficking into the cytosol and nucleus (Fig. 7, *A* and *B*) and propose that one or more transcriptional targets of Hap1 are responsible for mediating the mobilization of heme out of the mitochondria. We previously found that GTPases that control mitochondrial dynamics and ER contact sites are critical for regulating heme trafficking out of the mitochondria. Consequently, it is tempting to speculate that Hap1 might directly or indirectly regulate mitochondrial dynamics and contact sites to influence heme trafficking rates. Indeed, the heme senor Hap1 transcriptionally activates Hap4, which contains the activation domain of the heme-activated protein (HAP) complex, consisting of Hap2, Hap3, Hap4, and Hap5. The HAP complex regulates genes required for respiration, mitochondrial biogenesis and dynamics, and mitochondrial-ER contact sites (78, 79, 80). Future screening of deletion mutants of Hap1-target genes will reveal the factor(s) required for the Hap1-dependent mobilization of mitochondrial derived heme.

Overall, the studies herein provide new insights into determinants of heme availability, including heme buffering, the role of metabolically produced reactive oxygen species such as hydrogen peroxide in labilizing heme, and transcriptional control of heme trafficking dynamics. We anticipate that dynamic control of heme buffering capacity or peroxide-based redox signaling can control the lability of heme to in turn regulate downstream targets that are allosterically regulated by heme.

## Experimental Procedure

### Yeast strains, transformations, and growth conditions

*S. cerevisiae* strains used in this study were derived from strain S288C, including BY4741 (MATa, *his3*Δ1, *leu2*Δ0, *met15*Δ0, *ura3*Δ0) and FY2607 (*GAL2*^+^ MATa, *ura3Δ0*, *his3Δ200*, *leu2Δ0*, *lys2-128δ*, *hap1*^TY1^). BY4741 strains *ctt1*Δ*::KanMX4*, *hmx1*Δ*::KanMX4*, *yhb1*Δ*::KanMX4*, and *tdh3*Δ::*KanMX4* were obtained from the yeast gene deletion collection (Thermo-Fisher Scientific) and confirmed by sequencing the barcodes flanking the *KANMX4* deletion cassette. BY4741 DH001b-3 (BY4741 *hem1*Δ*::HIS3*) was previously reported (21). Strains FY2607, FY2609 (FY2607 *HAP1*), FY2611 (FY2607 hap1Δ0::*KanMX4*) and FY2613 (FY2607 myc-*HAP1*) were kindly provided by Dr Fred Winston (Harvard Medical School) and previously reported (43). The BY4741 GFP-tagged ORF collection, which was originally available from Thermo Scientific but no longer commercially available, was purchased from the High Throughput Analysis Lab of the Chemistry of Life Processes Institute at Northwestern University. Strains were confirmed by immunoblotting using α-GFP antibodies (Genetex; Cat # GTX30738; 1:5000 dilution) and sequencing.

Yeast Transformations were performed using the lithium acetate procedure (81). Strains were maintained at 30 °C on either enriched yeast extract (1%) and peptone (2%) based media supplemented with 2% glucose (YPD), or synthetic complete (SC) media with 2% glucose or SC media with the appropriate nutritional dropout mixture to maintain selection (Sunrise Science Products). Cells cultured on solid media plates were done so with YPD or SC media supplemented with 2% agar. Selection for yeast strains containing the KanMX4 marker was done on YPD agar plates supplemented with G418 (200 μg/ml). Culturing of *hem1*Δ cells required supplementing YPD or SC media with 50 μg/ml of 5-aminolevulinic acid (5-ALA) or 15 mg/L of ergosterol and 0.5% Tween-80 (21). All liquid cultures were maintained at 30 °C and shaken at 220 rpm.

### Plasmids

Hap1 transcriptional activity reporter plasmids were generated by engineering constructs in which eGFP is driven by the *CYC1* promoter (pMW003;*CYC1*^pr^-eGFP) or mKATE2 is driven by the *HMG2* promoter (pMK002; *HMG2*^pr^-mKATE2). The 5′ untranslated region of *CYC1* (−369 to −1 relative to the *CYC1* translational start site (82, 83)), flanked by a 5′ SpeI and 3′ BamHI restriction sites, was amplified from BY4741 yeast genomic DNA using PCR. The PCR product was digested and ligated into pRS415, generating pMW002. The eGFP gene, flanked by 5′ BamHI and 3′ HindIII restriction sites, was separately amplified from pRH003-1, which is a p425-GPD plasmid containing eGFP subcloned into the 5′ BamHI and 3′ HindIII sites of the multiple cloning site. The PCR product was digested and ligated between the BamHI and HindIII sites of pMW002, resulting in pMW003 (37).

The 5′ untranslated region of *HMG2* (−614 to −1 relative to the *HMG2* translational start site (84)) flanked by a 5′ EagI and 3′ XbaI restriction sites, was amplified from BY4741 yeast genomic DNA using PCR. The PCR product was digested and ligated into pRS316, generating pMW050. The mKATE2 gene, flanked by 5′ XbaI and 3′ BamHI restriction sites, was separately amplified from pDH013, which is a p415-GPD plasmid containing the HS1 heme sensor (21, 55). The PCR product was digested and ligated between the 5′ XbaI and 3′ BamHI sites of pMW050, resulting in pMK001. The 3′ untranslated region of *HMG2* that contains the terminator (+6 to +328 relative to the translational stop codon of *HMG2*) flanked by 5′ BamHI and 3′ EcoRI restriction sites was amplified by PCR. The PCR product was digested and ligated between the BamHI and EcoR1 sites of pMK001, generating pMK002.

The heme sensor plasmids utilized in this study were all previously reported (21, 56, 85) and include: pDH013 (p415-GPDpr-yHS1; Addgene Plasmid #159150), pRH021 (p415-GPDpr-yHS1-M7A; Addgene plasmid #159151), pJA010 (p415-GPDpr-HS1-M7A,H102A; Addgene Plasmid #159152), pMW016-2 (p415-GPDpr-mito-yHS1; Addgene Plasmid #159153), pAF001-3 (p415-GPDpr-mito-yHS1-M7A; Addgene Plasmid #159164), pAF003-4 (p415-GPDpr-mito-yHS1-M7A, H102A; Addgene Plasmid #159165), pDH046 (p415-GPDpr-nuc-yHS1; Addgene Plasmid #159166), HS1-M7A-NLS (p415-GPDpr-nuc-yHS1-M7A; Addgene Plasmid #159167), pCM009 (p415-GPDpr-nuc-yHS1-M7A, H102A; Addgene Plasmid #159168), or an empty vector plasmid (pRS415-GPD; Addgene Plasmid #183940). The Hap1 expression plasmids utilized in this study were all previously reported (42) and summarized in Figure 4*A*.

### Measurement of steady-state free heme

To measure steady-state free heme, triplicate yeast strains expressing cytosolic, mitochondrial, or nuclear-targeted HS1, HS1-M7A, HS1-M7A,H102A, or empty vector (EV) were cultured in 10 ml SC-LEU media, without or with the doses of succinylacetone indicated in the figure legends, for ∼14 to 16 h to mid-exponential phase (OD_600_ ≈ 1–2). After culturing, cells were collected, washed in water, and resuspended in phosphate-buffered saline (PBS) solution to a density of 5 OD_600_ units per ml. 200 μl of the cell suspension, corresponding to 1 OD_600_ units or 2 × 10^7^ cells, was used to measure eGFP (excitation 488 nm, emission 510 nm) and mKATE2 (excitation 588, emission 620) fluorescence. Background autofluorescence of cells expressing the empty vector was recorded and subtracted from the eGFP and mKATE2 sensor fluorescence values before calculating the eGFP/mKATE2 ratio (*R*). Fluorescence was recorded on a Synergy Mx multi-modal plate reader using black Greiner Bio-One flat-bottom fluorescence plates.

(1) $$\left[\%\;\text{Bound}\right]=\left(\frac{R-R_{\min}}{R_{\max}-R_{\min}}\right)*100$$

eGFP/mKATE2 fluorescence ratios (*R*) were converted to fractional sensor heme occupancies using Equation 1. The fractional saturation of the heme sensor (% Bound) is calculated by considering the sensor *R*-value relative to when it is 0% (*R*_min_) or 100% (*R*_max_) bound. *R*_max_, or the eGFP/mKATE2 fluorescence ratio associated with sensor saturation, was determined using an *in situ* calibration in which the eGFP and mKATE2 fluorescence was recorded after permeabilization of cells with digitonin (for cytosolic sensors) or solubilizing spheroplasts with Triton X-100 (for nuclear and mitochondrial sensors) and incubation with 50 μM hemin chloride and 1 mM ascorbate as previously described in detail (85). Briefly, 5 OD_600_/ml of cells were resuspended in PBS with 100 μg/ml of digitonin, 1 mM ascorbate, and 50 μM hemin chloride. After a 30-min incubation at 30 °C, cells were harvested, washed, and resuspended in PBS buffer prior to recording fluorescence by plate reader.

For mitochondrial or nuclear sensors, *R*_max_ was determined by digesting the yeast cell wall with Zymolyase, followed by incubation of yeast spheroplasts with 1.0% Triton-X100 to permeabilize membranes, and finally incubation the cell suspension with 1 mM ascorbate and 50 μM hemin chloride. Cells were spheroplasted by first incubating them in a “softening buffer” (100 mM Tris-HCl, pH = 9.4, 10 mM DTT) at a concentration of 10 OD_600_/ml of cells at at room temperature with occasional swirling for 30 to 60 min. Following this, cells were washed and resuspended in “sorbitol buffer” containing 50 mM Tris-HCl, pH 7.4, 1.2 M Sorbitol. Spheroplasting was initiated by adding 0.5 mg/ml of 100-T Zymolyase and incubating the suspension at 30 °C for 45 min with periodic inversion. Following this incubation period, the cells were washed in the sorbitol buffer and finally resuspend in the sorbitol buffer containing 1% Triton X-100, 1 mM ascorbate, and 50 μM hemin chloride at a final cell concentration of 5 OD_600_/ml. This suspension was incubated for 30 min at 30 °C. After this incubation period, the cell pellet was washed and resuspended in sorbitol buffer containing 1 mM ascorbate at a final concentration of 5 OD_600_/ml prior to recording of fluorescence by plate reader.

In BY4741 *HAP1*^TY1^ or *hap1*Δ cells, HS1 is quantitatively saturated in all compartments; *R*_max_ values for HS1 before and after *in situ* calibration are nearly identical. Consequently, parallel cultures of BY4741 *HAP1*^TY1^ cells expressing HS1 were also used to determine *R*_max_ *in lieu* of the *in situ* calibration in some experiments.

*R*_min_, or the eGFP/mKATE2 fluorescence ratio associated with *apo* sensor, was determined by recording eGFP and mKATE2 fluorescence in parallel cultures that were treated with 500 μM succinylacetone, from *hem1*Δ cells expressing the sensors, or from strains expressing the HS1-M7A, H102A sensor variant that cannot bind heme (85).

Free heme concentrations were estimated using Equation 2, where *α* is the fractional heme occupancy determined from Equation 1, and the *K*_D_ is the HS1-M7A sensor heme dissociation constant (85).

(2) $$\left[\text{Heme}\right]=\frac{\alpha \times K_{D}}{100-\alpha }$$

### Measurement of heme trafficking dynamics

Inter-compartmental heme trafficking rates were monitored as previously described (56, 85). Briefly, triplicate HS1-expressing cells were cultured with or without 500 μM succinylacetone (SA) (Sigma-Aldrich) in 10 ml SCE-LEU media in 50 ml conical tubes. Cultures were seeded at an initial density of 0.01 to 0.03 OD_600_/ml and grown for 14 to 16 h at 30 °C, shaking at 220 rpm until cells reached a density of OD_600_/ml between one and 2. After culturing, 10 OD_600_ units of cells (2 × 10^8^ cells) were collected, washed twice with 5 ml of sterile ultrapure water, and resuspended in 10 ml of fresh SC-LEU media in 50 ml conical tubes to give a density of 1 OD_600_/ml. The *HAP1*^TY1^ cells that were pre-cultured without succinylacetone provided reference values for eGFP/mKATE2 fluorescence ratios associated with maximal heme loading of HS1 (*R*_max_ values). The SA-conditioned cells were split into two 5 ml fractions. One fraction was treated with 500 μM SA to give eGFP/mKATE2 fluorescence ratios associated with minimal heme loading of HS1 (*R*_min_ values). The other fraction was not treated with SA so that heme synthesis could be re-initiated to give compartment-specific heme trafficking rates. HS1 fluorescence was monitored over a 4-h period by taking 0.5 ml aliquots at the indicated times from a 5 ml culture (initial density of 1 OD_600_/ml) shaking at 220 RPM at 30 °C. The 0.5-ml suspensions were washed and resuspended in 200 μl of PBS to a final density of 2.5 OD_600_/ml and eGFP and mKATE2 fluorescence were recorded. Sensor eGFP/mKATE2 ratios (*R*) in cells that had heme synthesis re-initiated after a period of SA-mediated heme depletion were converted to fractional heme occupancies using Equation 1. For each time point, *R*_max_ was derived from HS1 in *HAP1*^TY1^ cells that were continuously cultured without SA and *R*_min_ was derived from HS1 in the indicated strains cultured continuously with SA.

### Quantification of total cellular heme

Measurements of total heme were accomplished using a fluorometric assay designed to measure the fluorescence of protoporphyrin IX upon the release of iron from heme as previously described (56, 85). For all total heme measurements, ∼1 × 10^8^ cells were harvested, washed in sterile ultrapure water, and resuspended in 500 μl of 20 mM oxalic acid and stored in a closed box at 4 °C overnight (16–18 h). Next, an equal volume (500 μl) of 2 M oxalic acid was added to the cell suspensions in 20 mM oxalic acid. The samples were split, with half the cell suspension transferred to a heat block set at 95 °C and heated for 30 min and the other half of the cell suspension kept at room temperature (∼25 °C) for 30 min. All suspensions were centrifuged for 2 min on a table-top microfuge at 21,000*g* and the porphyrin fluorescence (ex: 400 nm, em: 620 nm) of 200 μl of each sample was recorded on a Synergy Mx multi-modal plate reader using black Greiner Bio-one flat bottom fluorescence plates. Heme concentrations were calculated from a standard curve prepared by diluting 500 to 1500 μM hemin chloride stock solutions in 0.1 M NaOH into ultrapure water, which was then added back to extra cell samples as prepared above. To calculate heme concentrations, the fluorescence of the unboiled sample (taken to be the background level of protoporphyrin IX) is subtracted from the fluorescence of the boiled sample (taken to be the free base porphyrin generated upon the release of heme iron). The cellular concentration of heme is determined by dividing the moles of heme determined in this fluorescence assay and dividing by the number of cells analyzed, giving moles of heme per cell, and then converting to a cellular concentration by dividing by the volume of a yeast cell, taken to be 50 fl (21).

To measure heme synthesis rates, exponential-phase cells were pre-conditioned with 500 μM SA for 14 to 16 h in SCE-LEU media as described above for the heme trafficking dynamics assay. Following this, cells were washed and resuspended in media lacking SA and aliquots of cells were harvested and washed for total heme quantification as described above.

### Quantification of total cytosolic and mitochondrial heme

Mitochondrial and cytosolic extracts were isolated using yeast mitochondrial (Cat #K259-50) isolation kits from BioVision according to the manufacturer’s instructions as previously described (56). Triplicate 50 ml cultures of BY4741 WT yeast in SC-LEU in 250 ml Erlenmeyer flasks were grown to a density of OD_600_/ml = 1.0 (2 × 10^7^ cells/ml). 1 × 10^9^ cells were harvested, washed in ultrapure water, and resuspended in “Buffer A” containing 10 mM DTT for 30 min at 30 °C with occasional gentle agitation. Cells were then harvested by centrifugation at 1500*g* for 5 min at room temperature. Cells were then resuspended in 2 ml of “Buffer B” containing manufacturer-provided Zymolyase and incubated at 30 °C with occasional gentle inversion. Spheroplast formation was monitored by diluting 10 uL of the cell suspension into ultrapure water and monitoring the decrease in OD_600nm_ such that it was > 80% less than the initial value, which typically took 30 to 60 min. Spheroplasts were harvested by centrifugation at 1500*g* for 5 min, resuspended in “Homogenization Buffer” containing a protease inhibitor cocktail, and lysed with 15 strokes of a Dounce Homogenizer on ice. The lysate was centrifuged at 600*g* for 5 min at 4 °C to remove cell debris. The supernatant, which contained the mitochondria, was again centrifuged for 5 min at 600*g* to remove additional debris. Finally, mitochondria was harvested by centrifugation at 12,000*g* for 10 min at 4 °C. The mitochondrial pellet was resuspended in 200 μl of “Storage Buffer” and the supernatant associated with the mitochondrial pellet was collected as the “cytosolic” extract (∼2 ml).

Immunoblotting for cytosolic and mitochondrial marker proteins confirmed the isolation of these respective extracts. The total spheroplast fraction (total cell), mitochondrial fraction, and the post-mitochondrial fraction (supernatant from the 12,000*g* centrifugation step which is taken to be the cytosol) were electrophoresed on a 14% tris-glycine SDS-PAGE gel and immunoblotted using α-PGK1 (Life Technologies; Cat # PA528612; 1:5000 dilution), a cytosolic marker protein, or α-porin (Life Technologies; Cat # 459500; 1:5000 dilution), a mitochondrial marker protein. A goat α-rabbit secondary antibody conjugated to a 680 nm emitting fluorophore (VWR/Biotium; Cat # 89138-520; 1:10,000 dilution) was used to probe for PGK1 and a goat α-mouse secondary antibody conjugated to a 680 nm emitting fluorophore (VWR/Biotium; Cat # 89138-516; 1:10,000 dilution) was used to probe for Porin. All gels were imaged on a LiCOR Odyssey Infrared imager (21, 86).

The heme content of “cytosolic” and “mitochondrial” fractions was analyzed using the oxalic acid method described above. In this case, 0.5 ml of the cytosolic fraction and 0.1 ml of the mitochondrial fraction were used as input for the total heme measurements. The heme content associated with the cytosolic and mitochondrial fractions were converted to molar concentrations of heme in the cytosol or mitochondria using Equation 3.

(3) $${\left[\text{Heme}\right]}_{cyt/mito}=\frac{\frac{m_{\text{Heme}}}{{1\times 10}^{9}\text{cells}}}{V_{cyt/mito}}$$

The moles of heme (*m*) associated with the cytosolic or mitochondrial fraction were determined by estimating the concentration of heme from the standard curve and multiplying this value by the total volume of cytosolic or mitochondrial extract. The moles of cytosolic or mitochondrial heme per cell was then obtained by dividing the *m*-value by the total number of cells used to isolate the cytosolic or mitochondrial extracts (∼1 × 10^9^ cells). Lastly, the moles of cytosolic or mitochondrial heme per cell were converted to a molar concentration of cytosolic or mitochondrial heme by dividing through by the volume (*V*) of the yeast cytosol (35 fl) or mitochondria (0.5 fl).

### Hap1 reporter activity

The indicated yeast strains expressing the Hap1 transcriptional reporters, pMW003 (*CYC1*^pr^–eGFP) or pMK002 (*HMG2*^pr^-mKATE2), were cultured in 10 ml SCE-LEU media for ∼14 to 16 h to a density of 1 to 2 OD_600_/ml. Cells were resuspended in PBS to a concentration of 10 OD_600_/ml and 200 μLs of this suspension was used to measure eGFP or mKATE2 fluorescence. Background autofluorescence of empty vector cells not expressing eGFP of mKATE2 was recorded and subtracted from the eGFP and mKATE2-containing Hap1 reporter strains.

### Immunoblotting

Measurements of the relative protein expression levels of catalase (Ctt1), GAPDH, or various GFP-tagged heme proteins were assessed by immunoblotting. Briefly, the indicated yeast strains were cultured in 10 ml SCE media for 14 to 16 h to mid-exponential phase to an OD_600_/ml value of 1. 10 OD_600_ units of cells were washed in ultrapure water, harvested, and lysed in two pellet volumes of lysis buffer (10 mM sodium phosphate, 50 mM sodium chloride, 5 mM EDTA, 0.1% Triton X-100, 1 mM PMSF and a protease inhibitor cocktail (GBiosciences) as described previously (87, 88). Lysis was achieved at 4 °C using one pellet volume of zirconium oxide beads and a bead beater (Bullet Blender, Next Advance) on a setting of eight for 3 min [84]. Lysate protein concentrations were determined by the Bradford method (Bio-rad) and 20 to 80 μg of lysate was employed for SDS-PAGE analysis using 14% tris-glycine gels (Invitrogen). After transfer to nitrocellulose membranes, membranes were blocked with the LICOR protein-free blocking buffer and probed using the anti-Ctt1 antibody (21) (1:1000 dilution; overnight probing at 4 °C of 75–100 μg of protein lysate), anti-GAPDH antibody (Genetex; Cat # GTX627408; 1:10,000 dilution), α-GFP antibody (Genetex; Cat # GTX30738; 1:5000 dilution) or LICOR total protein stain as a loading control. A goat α-rabbit secondary antibody conjugated to a 680 nm emitting fluorophore (VWR/Biotium; Cat # 89138-520; 1:10,000 dilution) was used to probe for Ctt1 and GFP-tagged proteins and a goat α-mouse secondary antibody conjugated to a 680 nm emitting fluorophore (VWR/Biotium; Cat # 89138-516; 1:10,000 dilution) was used to probe for GAPDH. All gels were imaged on a LiCOR Odyssey Infrared imager (21, 86).

### Measurements of catalase activity

The activity of yeast catalase was measured following the methods described previously (37). To detect the catalase activity, 15 μg protein lysate was analyzed by native PAGE on a 10% Tris-glycine gel (Invitrogen). After electrophoresis, an in-gel activity stain was utilized to measure catalase activity. In brief, the catalase staining solution was prepared by mixing one part dopamine (20 mg ml^−1^) and one part para-phenylenediamine (3.5 mg ml^−1^) in 0.2 M potassium phosphate buffer, pH 8, 1 part 15% H_2_O_2_, and two parts DMSO in the order listed. The staining solution was added directly to the gel and allowed to stain for 2 min, followed by imaging.

### Equilibrium modeling of heme

The various assumptions and details underlying the equilibrium model and the heme dissociation constants and reduction potentials of yeast hemoproteins are outlined in the Supporting Information. The median copy numbers of known heme-dependent proteins and heme homeostatic factors were obtained from reference (57) and listed in Tables 1 and 2. The median copy numbers of the “cytosolic” heme-dependent proteins (Table 1) were converted to molar concentrations by dividing through by Avogadro’s number and the volume of the yeast cytosol, taken to be 35 fl (54) (Tables S1–S3). The molar concentrations and assumed apparent heme dissociation constants discussed in the results section were then used as input in ChemEQL version 3.2.1, a program to calculate chemical speciation equilibria (89), to calculate free heme. Free heme calculated using ChemEQL was converted to heme sensor occupancy (α) by algebraic rearrangement of Equation 2. Table S3 outlines the matrix of input proteins, concentrations, and apparent dissociation constants used to parameterize calculations of free heme presented in the manuscript.

### Statistics

All experimental trials include at least three biological replicates. Appropriate analyses of variance (ANOVA) were conducted using Graph Pad Prism software (version 11.0.1) and described in the figure legends.

## Data availability

All data are included in this publication or in the Supporting Information (21, 23, 24, 39, 55, 66, 75, 90, 91, 92, 93, 94, 95, 96, 97, 98, 99, 100, 101, 102, 103, 104, 105, 106, 107, 108, 109, 110, 111), including figures and tables. If additional information is desired, please contact Amit Reddi at amit.reddi@chemistry.gatech.edu.

## Supporting information

This article contains Supporting Information (21, 23, 24, 39, 55, 66, 75, 90, 91, 92, 93, 94, 95, 96, 97, 98, 99, 100, 101, 102, 103, 104, 105, 106, 107, 108, 109, 110, 111).

## Conflict of interest

The authors declare that they have no conflicts of interest with the contents of this article.
