# Supplementary material for: Regulation of free heme and heme buffering in Saccharomyces cerevisiae
Source: J Biol Chem. 2026 Sep 23;302(11):113603. doi: 10.1016/j.jbc.2026.113603 (PMC13722074; doi:10.1016/j.jbc.2026.113603)
Supplement: Supplementary file 1 [file mmc2.docx]

**Supporting Information**

**Regulation of Free Heme and Heme Buffering in *Saccharomyces cerevisiae***

Mathilda M. Willoughby^1^, Yibo Fu^1^, Courtney M. Moore^1^, Yungeun Lee^1^, Mauna S. Kareti^1^, Liushu Wu^1^, Li Zhang^2^, and Amit R. Reddi^1,3,4^*

*Corresponding Author

Author Affiliations:

^1^School of Chemistry and Biochemistry, Georgia Institute of Technology, Atlanta, GA 30332, USA

^2^ Department of Biological Sciences, The University of Texas at Dallas, Richardson, TX 75080, USA

^3^Parker Petit Institute for Bioengineering and Biosciences, Georgia Institute of Technology, Atlanta, GA 30332, USA

^4^School of Biological Sciences, Georgia Institute of Technology, Atlanta, GA 30332, USA

^*^Corresponding Author:

Amit R. Reddi

Georgia Institute of Technology

School of Chemistry and Biochemistry

901 Atlantic Drive, MC0400

Atlanta, GA 30332, USA

Phone: (404) 385-1428

E-mail address: amit.reddi@chemistry.gatech.edu

**Assumptions of the equilibrium model and outline of the heme dissociation constants and reduction potentials of yeast hemoproteins.**

**Oxidation state of free heme.** Our past work suggested that free heme in Baker’s yeast is predominantly in its reduced state (1). Indeed, the reduction potential of monomeric unbound aqueous heme is -50 mV vs. NHE (2, 3) and the cytosolic redox environment, which is maintained by the glutathione redox buffer, is relatively reducing (E_m_^Cytosol^ ~ -320 mV vs. NHE) (4). As a result, our equilibrium model assumes that free heme is reduced and, if oxidized heme dissociates from a protein, its reduction is rapid. Consequently, we assume that the heme sensor is measuring unbound reduced heme, which equilibrates with heme of either oxidation state from exchange labile binding sites (**Equation 1**).

**Parameters governing heme dissociation.** To estimate free heme, heme-protein dissociation constants and/or their reduction potentials are considered. If the resting oxidation state of heme in a protein is reduced, it is assumed that the concentration of free heme is governed by the ferrous heme dissociation constant, *K*_D_^II^ (**Equations 2 and 3**). If the resting oxidation state of heme in a protein is oxidized, the concentration of free heme is assumed to be governed by the product of *K*_D_^II^ and the equilibrium constant for heme reduction, *K*_eq_^red^, (**Equation 4**), which gives the overall apparent heme dissociation constant associated with heme reduction upon release, *K*_D_^’^ (**Equations 5 and 6**). If a protein was previously found to irreversibly bind ferric heme, or if the heme binding site is buried, the *K*_D_^III^ value is fixed to be 1 x 10^-14^ M, which essentially makes heme dissociation negligible.

$${Heme-Fe}^{2+}+L\underset{\leftrightarrow}{K_{D}^{\mathrm{II}}}{Heme-Fe}^{2+}-L \underset{\leftrightarrow}{K_{\mathrm{eq}}^{\mathrm{red}}} {Heme-Fe}^{3+}-L +e^{-}(Equation 1)$$

$${Heme-Fe}^{2+}+L\underset{\leftrightarrow}{K_{D}^{\mathrm{II}}} {Heme-Fe}^{2+}-L \left( Equation 2 \right)$$

$$K_{D}^{\mathrm{II}}=\frac{\left[ Heme-\mathrm{Fe}^{2+} \right][L]}{[Heme-\mathrm{Fe}^{2+}-L]}(Equation 3)$$

$$K_{\mathrm{eq}}^{\mathrm{red}}=\frac{\left[ Heme-\mathrm{Fe}^{2+}-L \right]}{\left[ Heme-\mathrm{Fe}^{3+}-L \right][e^{-}]}(Equation 4)$$

$${Heme-Fe}^{2+}+L\underset{\leftrightarrow}{K_{D}^{'}} {Heme-Fe}^{3+}-L+e^{-} (Equation 5)$$

$$K_{D}^{'}=\frac{\left[ Heme-\mathrm{Fe}^{2+} \right][L]}{\left[ Heme-\mathrm{Fe}^{3+}-L \right][e^{-}]}= K_{D}^{\mathrm{II}}\times K_{\mathrm{eq}}^{\mathrm{red}} (Equation 6)$$

**Estimating ferrous heme dissociation constants.** Given the general lack of biophysical data available for yeast heme proteins, their heme binding affinities and reduction potentials must be assumed based on characterization of similar or analogous proteins from other species. Moreover, since ferrous heme dissociation constants are rarely reported, they must be estimated using **Equation 7**, which is algebraically derived from a thermodynamic cycle that relates the reduction potentials of bound and free heme to the ferric and ferrous heme dissociation constants (3, 5).

$$\frac{K_{D}^{\mathrm{III}}}{e^{\frac{{nF[E}_{m}^{\mathrm{Bound}} - E_{m}^{\mathrm{Free}}]}{\mathrm{RT}}}} =K_{D}^{\mathrm{II}} (Equation 7)$$

In **Equation 7**, F is Faraday’s constant (96,485 C mol^-1^), R is the gas constant (8.314 J mol^-1^ K^-1^), T is temperature (298 K), *n* is the number of electrons involved in the redox process (*n* = 1), *E*_m_^Bound^ is the midpoint reduction potential of bound heme, and *E*_m_^Free^ is the midpoint reduction potential of free monomeric aqueous heme (taken to be -50 mV vs. NHE (2, 3)).

**Estimating apparent heme dissociation constants of yeast hemoproteins.** The heme proteins assumed to be reduced in their resting states and for which *K*_D_^II^ is expected to govern the concentration of free heme include hemoglobin-like protein YNL234W (6) and the tryptophan 2,3-dioxygenase (TDO)/indoleamine 2,3-dioxygenase (IDO) Bna2 (7). These proteins bind dioxygen as part of their function, which requires that heme iron is reduced in its resting state. We assume that YNL234W exhibits a ferric heme dissociation constant similar to that of the well characterized sperm whale myoglobin, *K*_D_^III^ = 1.0 x 10^-14^ M (8). Since other oxygen binding globins have relatively high potentials, *e.g.* myoglobin is ~+50 mV vs. NHE and hemoglobin is ~+150 mV vs. NHE (9), we will assume a reduction potential of ~+100 mV vs. NHE for YNL234W. Consequently, we can estimate a YNL234W *K*_D_^II^ value of 2.9 x 10^-17^ M using **Equation 7**.

With respect to Bna2, it was previously found that human heme dioxygenases indoleamine 2,3-dioxygenase (IDO) and tryptophan 2,3-dioxygenase (TDO) exist as a mixture of *apo* and *holo* states in cells (10, 11) and can readily dissociate and transfer heme to *apo*-myoglobin (12). Since the resting state of heme in these proteins is expected to be reduced (9), the reversibility and fractional heme occupancy of IDO/TDO suggest that its ferrous heme dissociation constant may be on the order of free heme measured in yeast and various non-erythroid mammalian cell lines, ~10 nM (1, 13-15). Thus, it is assumed that Bna2 has a ferrous heme dissociation constant of 10 nM.

The heme proteins assumed to be oxidized in their resting states and for which *K*_D_^’^ is expected to govern the concentration of free heme include flavohemoglobin (Yhb1), cytochrome *b*_5_ (Cyb5) or cytochrome *b*_5_ domain containing proteins (Irc21 and Scs7), cytosolic catalase (Ctt1), cytochrome P_450_ enzymes (Erg11 and Erg5), ferric iron reductases (Fre1-7), and the yeast NADPH oxidase (Yno1). It is assumed that the globin Yhb1 exhibits a ferric heme dissociation constant similar to sperm whale myoglobin, *K*_D_^III^ = 1.0 x 10^-14^ M (8). The resting state of heme in a flavohemoglobin is likely oxidized since it must accept electrons from a flavin as part of its mechanism. The Yhb1 heme reduction potential is expected to be similar to that of other flavohemoglobins, such as one from *Staphylococcus aureus*; -170 mV vs. NHE (16). Consequently, *K*_D_^II^ for Yhb1 can be estimated to be 1 x 10^-12^ M using **Equation 7** and *K*_D_^’^ is estimated to be 1.3 x 10^-15^ M.

It is assumed that yeast cytochrome *b*_5_ or any protein with a cytochrome *b*_5_ domain (Irc21, Cyb5, Scs7) exhibit similar *K*_D_^III^ values to that measured for human cytochrome *b*_5,_ 6.7 x 10^-13^ M (17, 18). The resting state of heme in cytochrome *b*_5_-like proteins is likely oxidized due to their roles in electron transfer. Since cytochrome *b*_5_ has a heme reduction potential of ~-100 mV vs. NHE (19), we estimate *K*_D_^II^ to be 4.7 x 10^-12^ M and K_D_’ to be 2.3 x 10^-10^ M.

In other cases, heme may be kinetically trapped as in catalase (18), precluding the determination of a heme dissociation constant. The yeast cytosolic catalase (Ctt1) ferric heme dissociation constant was therefore fixed to be 1 x 10^-14^ M, effectively making heme binding irreversible. Catalases have relatively low reduction potentials, -500 mV vs. NHE (20), and require that heme is oxidized in its resting state prior to peroxide coordination. Given the reduction potential, we can estimate *K*_D_^II^ to be 4 x 10^-7^ M and *K*_D_’ to be 1.4 x 10^-15^ M.

Similarly, heme may bind irreversibly to cytochrome P_450_ enzymes since their heme binding sites are buried. Indeed, prior studies found that when a ferric heme loaded cytochrome P_450_ was co-incubated with an equivalent of *apo* cytochrome *b*_5_, < 5% of the heme transferred to *apo* cytochrome *b*_5_ (21), suggesting that the ferric heme dissociation constant is < 2 x 10^-15^ M. Consequently, it is assumed that the cytochrome P_450_ enzymes (Erg11 and Erg5) have ferric heme dissociation constants of ~2 x 10^-15^ M. Like catalases and peroxidases, cytochrome P_450_ enzymes have relatively low substrate-free reduction potentials, *e.g.* -427 mV vs. NHE for cytochrome P_450_^BM3^ (22), and require heme is oxidized in its resting state. Assuming the same low potential for Erg11 and Erg5, *K*_D_^II^ is estimated to be ~4.6 x 10^-9^ M and *K*_D_’ to be 2.8 x 10^-16^ M.

Due to heme burial in ferric iron reductases (Fre1-7) and NADPH oxidases (Yno1), we assume heme binds irreversibly and fixed the *K*_D_^III^ value to be 1 x 10^-14^ M. The resting state of heme in these enzymes is expected to be oxidized due to its function in passing electrons. Ferric iron reductases exhibit relatively high heme reduction potentials, ~+150 – +200 mV for the high potential site and ~-20 mV – +60 mV for the low potential site (23, 24). By averaging the mid-range potential for the two heme sites, +175 mV and +20 mV, we calculate a composite potential of ~100 mV. Assuming this heme potential for the Fre proteins, *K*_D_^II^ is estimated to be 2.9 x 10^-17^ M and K_D_’ to be 1.4 x 10^-15^ M. The heme reduction potential in NADPH oxidases is -245 mV vs. NHE (25, 26). Assuming this heme potential for Yno1, *K*_D_^II^ is estimated to be 2 x 10^-11^ M and *K*_D_’ to be 1.4 x 10^-15^ M.

GAPDH selectively binds ferric heme over ferrous heme (27), making *K*_D_’ govern heme dissociation. The *K*_D_^III^ value was previously found to be 130 nM, which is the average *K*_D_ value determined from direct titration or estimated from association and dissociation rates (*K*_D_ = 24 nM (27); *K*_D_ = 68 nM (28); K_D_ = 150 nM (29); *K*_D_ = 19 nM (27); *K*_D_ = 390 nM (27)). Given the reduction potential of the heme-GAPDH complex, -197 mV vs. NHE (27), *K*_D_^II^ is estimated to be 4.0 x 10^-5^ M and *K*_D_’ to be 1.9 x 10^-8^ M.

**Supplemental Figure 1.** Alternative assumptions used to calculate free heme and heme sensor occupancy. (**A**) Prediction of heme sensor occupancy as a function GAPDH concentration for 5 and 10 μM total cytosolic heme. The concentrations and apparent heme dissociation constants used to parameterize the calculation are outlined in **Table S3** for WT cells, with the exception that GAPDH concentration is varied between 1 – 50 μM, to assess its impact on free heme and sensor occupancy. (**B**) Prediction of sensor heme occupancy for total concentrations of heme between 5 and 10 μM as a function of various apparent heme dissociation constants. The concentrations used to parameterize the calculation are outlined in **Table S3** for WT cells with and without the GAPDH buffer. In this case, however, the apparent heme dissociation constants *K*_D_’ or K_D_^II^ were fixed as 10 fM, 1 nM, 10 nM, or 100 nM. (**C**) Prediction of sensor heme occupancy for total concentrations of heme between 5 and 10 μM as a function of free heme oxidation state. The concentrations used to parameterize the calculation are outlined in **Table S3** for WT cells with and without the GAPDH buffer. When assuming heme is reduced (red), the assumptions outlined in Table S3 were used and heme sensor occupancy was calculated assuming the HS1-M7A K_D_^II^ values of 25 nM. When assuming that free heme is oxidized (ox), *K*_D_^III^ values from **Table S3** are used and heme sensor occupancy was calculated assuming the HS1-M7A K_D_^III^ values of 1 μM.

**Supporting Information References**

1.	Hanna, D. A., Harvey, R. M., Martinez-Guzman, O., Yuan, X., Chandrasekharan, B., Raju, G. *et al.* (2016) Heme dynamics and trafficking factors revealed by genetically encoded fluorescent heme sensors *Proc Natl Acad Sci U S A* **113**, 7539-7544

2.	Conant, J. B., and Tongberg, C. O. (1930) The oxidation-reduction potentials of hemin and related substances: I. The potentials of various hemins and hematins in the abscence and presence of pyridine *Journal of Biological Chemistry* **86**, 733-741

3.	Reddi, A. R., Reedy, C. J., Mui, S., and Gibney, B. R. (2007) Thermodynamic investigation into the mechanisms of proton-coupled electron transfer events in heme protein maquettes *Biochemistry* **46**, 291-305

4.	Hu, J., Dong, L., and Outten, C. E. (2008) The redox environment in the mitochondrial intermembrane space is maintained separately from the cytosol and matrix *J Biol Chem* **283**, 29126-29134

5.	Reedy, C. J., Elvekrog, M. M., and Gibney, B. R. (2008) Development of a heme protein structure-electrochemical function database *Nucleic Acids Res* **36**, D307-313

6.	Sartori, G., Aldegheri, L., Mazzotta, G., Lanfranchi, G., Tournu, H., Brown, A. J. *et al.* (1999) Characterization of a new hemoprotein in the yeast Saccharomyces cerevisiae *J Biol Chem* **274**, 5032-5037

7.	Panozzo, C., Nawara, M., Suski, C., Kucharczyka, R., Skoneczny, M., Becam, A. M. *et al.* (2002) Aerobic and anaerobic NAD+ metabolism in Saccharomyces cerevisiae *FEBS Lett* **517**, 97-102

8.	Hargrove, M. S., Barrick, D., and Olson, J. S. (1996) The association rate constant for heme binding to globin is independent of protein structure *Biochemistry* **35**, 11293-11299

9.	Millett, E. S., Efimov, I., Basran, J., Handa, S., Mowat, C. G., and Raven, E. L. (2012) Heme-containing dioxygenases involved in tryptophan oxidation *Curr Opin Chem Biol* **16**, 60-66

10.	Thomas, S. R., Salahifar, H., Mashima, R., Hunt, N. H., Richardson, D. R., and Stocker, R. (2001) Antioxidants inhibit indoleamine 2,3-dioxygenase in IFN-gamma-activated human macrophages: posttranslational regulation by pyrrolidine dithiocarbamate *J Immunol* **166**, 6332-6340

11.	Biswas, P., Dai, Y., and Stuehr, D. J. (2022) Indoleamine dioxygenase and tryptophan dioxygenase activities are regulated through GAPDH- and Hsp90-dependent control of their heme levels *Free Radic Biol Med* **180**, 179-190

12.	Nelp, M. T., Kates, P. A., Hunt, J. T., Newitt, J. A., Balog, A., Maley, D. *et al.* (2018) Immune-modulating enzyme indoleamine 2,3-dioxygenase is effectively inhibited by targeting its apo-form *Proc Natl Acad Sci U S A* **115**, 3249-3254

13.	Hanna, D. A., Hu, R., Kim, H., Martinez-Guzman, O., Torres, M. P., and Reddi, A. R. (2018) Heme bioavailability and signaling in response to stress in yeast cells *J Biol Chem* **293**, 12378-12393

14.	Song, Y., Yang, M., Wegner, S. V., Zhao, J., Zhu, R., Wu, Y. *et al.* (2015) A Genetically Encoded FRET Sensor for Intracellular Heme *ACS Chem Biol* **10**, 1610-1615

15.	Leung, G. C.-H., Fung, S. S.-P., Gallio, A. E., Blore, R., Alibhai, D., Raven, E. L. *et al.* (2021) Unravelling the mechanisms controlling heme supply and demand *Proceedings of the National Academy of Sciences* **118**, e2104008118

16.	Goncalves, V. L., Nobre, L. S., Vicente, J. B., Teixeira, M., and Saraiva, L. M. (2006) Flavohemoglobin requires microaerophilic conditions for nitrosative protection of Staphylococcus aureus *FEBS Lett* **580**, 1817-1821

17.	Falzone, C. J., Wang, Y., Vu, B. C., Scott, N. L., Bhattacharya, S., and Lecomte, J. T. (2001) Structural and dynamic perturbations induced by heme binding in cytochrome b5 *Biochemistry* **40**, 4879-4891

18.	Gilep, A. A., Guryev, O. L., Usanov, S. A., and Estabrook, R. W. (2001) Apo-cytochrome b5 as an indicator of changes in heme accessability: preliminary studies with cytochrome P450 3A4 *J Inorg Biochem* **87**, 237-244

19.	Rivera, M., Seetharaman, R., Girdhar, D., Wirtz, M., Zhang, X., Wang, X. *et al.* (1998) The reduction potential of cytochrome b5 is modulated by its exposed heme edge *Biochemistry* **37**, 1485-1494

20.	Hosseinzadeh, P., and Lu, Y. (2016) Design and fine-tuning redox potentials of metalloproteins involved in electron transfer in bioenergetics *Biochim Biophys Acta* **1857**, 557-581

21.	Yamazaki, H., Shimada, T., Martin, M. V., and Guengerich, F. P. (2001) Stimulation of cytochrome P450 reactions by apo-cytochrome b5: evidence against transfer of heme from cytochrome P450 3A4 to apo-cytochrome b5 or heme oxygenase *J Biol Chem* **276**, 30885-30891

22.	Rajakumara, E., Saniya, D., Bajaj, P., Rajeshwari, R., Giri, J., and Davari, M. D. (2022) Hijacking Chemical Reactions of P450 Enzymes for Altered Chemical Reactions and Asymmetric Synthesis *Int J Mol Sci* **24**,

23.	Berczi, A., Marton, Z., Laskay, K., Toth, A., Rakhely, G., Duzs, A. *et al.* (2023) Spectral and Redox Properties of a Recombinant Mouse Cytochrome b561 Protein Suggest Transmembrane Electron Transfer Function *Molecules* **28**,

24.	Cain, T. J., and Smith, A. T. (2021) Ferric iron reductases and their contribution to unicellular ferrous iron uptake *J Inorg Biochem* **218**, 111407

25.	Doussiere, J., Gaillard, J., and Vignais, P. V. (1999) The heme component of the neutrophil NADPH oxidase complex is a target for aryliodonium compounds *Biochemistry* **38**, 3694-3703

26.	Cross, A. R., Higson, F. K., Jones, O. T., Harper, A. M., and Segal, A. W. (1982) The enzymic reduction and kinetics of oxidation of cytochrome b-245 of neutrophils *Biochem J* **204**, 479-485

27.	Hannibal, L., Collins, D., Brassard, J., Chakravarti, R., Vempati, R., Dorlet, P. *et al.* (2012) Heme binding properties of glyceraldehyde-3-phosphate dehydrogenase *Biochemistry* **51**, 8514-8529

28.	Biswas, P., Palazzo, J., Schlanger, S., Jayaram, D. T., Islam, S., Page, R. C. *et al.* (2024) Visualizing mitochondrial heme flow through GAPDH in living cells and its regulation by NO *Redox Biol* **71**, 103120

29.	Sweeny, E. A., Singh, A. B., Chakravarti, R., Martinez-Guzman, O., Saini, A., Haque, M. M. *et al.* (2018) Glyceraldehyde-3-phosphate dehydrogenase is a chaperone that allocates labile heme in cells *J Biol Chem* **293**, 14557-14568
